# Epigenetic regulation of DNA repair gene program by Hippo/YAP1-TET1 axis mediates sorafenib resistance in HCC

**DOI:** 10.1007/s00018-024-05296-y

**Published:** 2024-07-05

**Authors:** Chunli Mo, Weixin You, Yipeng Rao, Zhenping Lin, Shuai Wang, Ting He, Huanming Shen, Xun Li, Rui Zhang, Boan Li

**Affiliations:** 1 State Key Laboratory of Cellular Stress Biology, Innovation Center for Cell Signaling Network, School of Life Sciences, Xiamen University, Xiamen, 361100 Fujian China; 2 The First Affiliated Hospital , of Xiamen University, Xiamen, 361100 Fujian China; 3 Department of Laboratory Medicine The First Affiliated Hospital, School of Medicine, Xiamen University, Xiamen, 361003 Fujian China; 4 Xiamen Cell Therapy Research Center, The First Affiliated Hospital, School of Medicine, Xiamen University, Xiamen, 361003 Fujian China; 5 Center for Precision Medicine, The First Affiliated Hospital of Xiamen University, Xiamen, China

**Keywords:** Hepatocellular carcinoma, 5-methylcytosine hydroxylase, Chemotherapy resistance, DNA damage response, Methylation modification

## Abstract

Hepatocellular carcinoma (HCC) is a malignancy that occurs worldwide and is generally associated with poor prognosis. The development of resistance to targeted therapies such as sorafenib is a major challenge in clinical cancer treatment. In the present study, Ten-eleven translocation protein 1 (TET1) was found to be highly expressed in sorafenib-resistant HCC cells and knockdown of TET1 can substantially improve the therapeutic effect of sorafenib on HCC, indicating the potential important roles of TET1 in sorafenib resistance in HCC. Mechanistic studies determined that TET1 and Yes-associated protein 1 (YAP1) synergistically regulate the promoter methylation and gene expression of DNA repair-related genes in sorafenib-resistant HCC cells. RNA sequencing indicated the activation of DNA damage repair signaling was extensively suppressed by the TET1 inhibitor Bobcat339. We also identified TET1 as a direct transcriptional target of YAP1 by promoter analysis and chromatin-immunoprecipitation assays in sorafenib-resistant HCC cells. Furthermore, we showed that Bobcat339 can overcome sorafenib resistance and synergized with sorafenib to induce tumor eradication in HCC cells and mouse models. Finally, immunostaining showed a positive correlation between TET1 and YAP1 in clinical samples. Our findings have identified a previously unrecognized molecular pathway underlying HCC sorafenib resistance, thus revealing a promising strategy for cancer therapy.

**Graphical Abstract:**

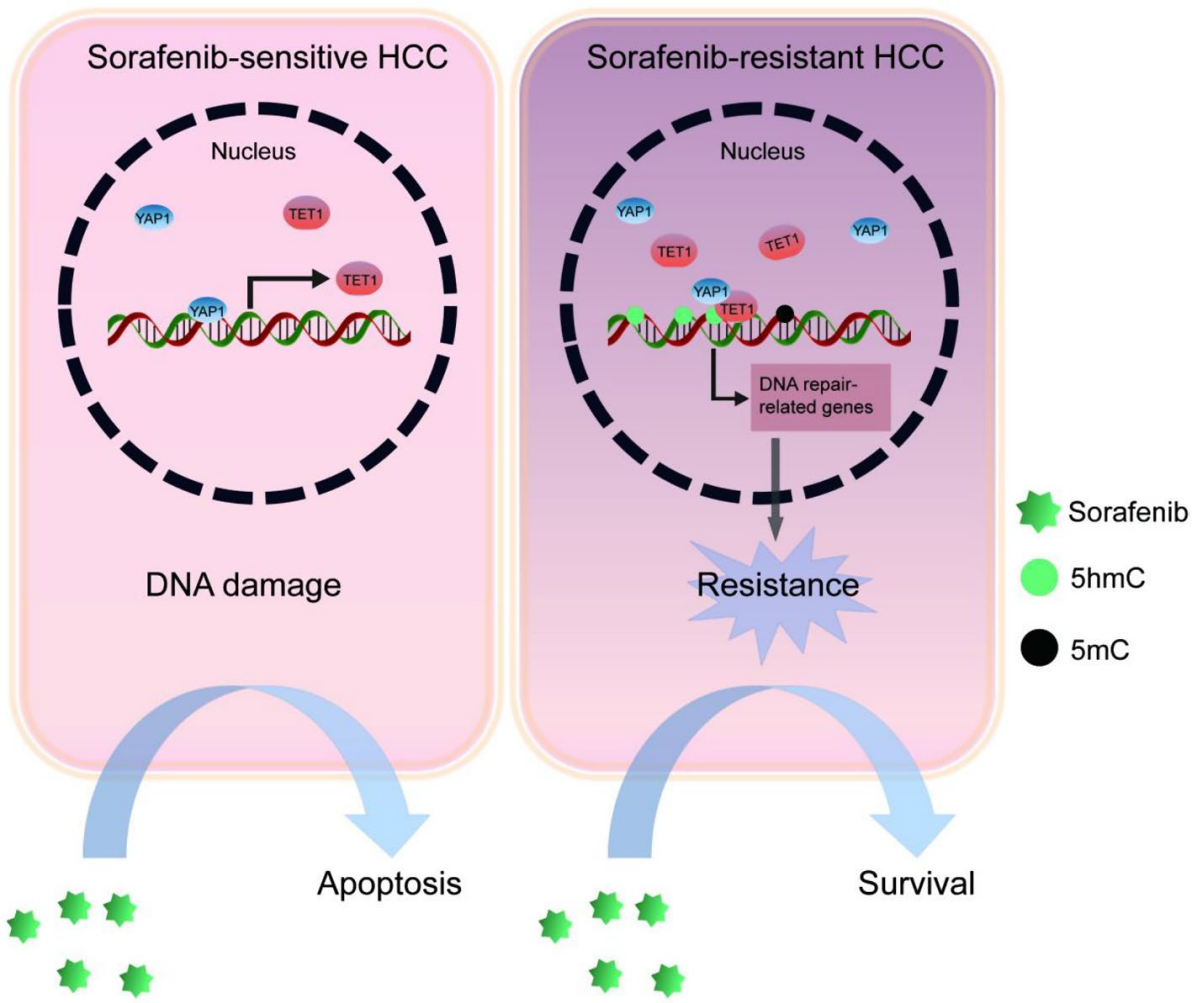

**Supplementary Information:**

The online version contains supplementary material available at 10.1007/s00018-024-05296-y.

## Introduction

HCC is one of the prevalent cancers worldwide. Sorafenib, a first-line targeted therapy, is a multi-kinase inhibitor used for the treatment of advanced HCC, and it has been shown that the median survival time of patients was extended from 7.9 to 10.7 months [1, 2]. However, the development of resistance limits the effectiveness of sorafenib. Sorafenib resistance in HCC usually develops within 6 months after treatment [3]. Despite this, the mechanisms underlying sorafenib resistance are still poorly understood. Therefore, there is an urgent need for further research into the molecular mechanisms of sorafenib resistance and new therapeutic strategies to overcome this challenge.

The DNA repair pathway enables normal and cancer cells to survive DNA damage induced after therapies [4]. Cancer cells often possess abnormal DNA repair capacity, which helps them evade DNA damage induced by treatments and leads to the development of treatment resistance. Aberrant activation of DNA repair pathways can repair therapy-induced DNA damage, allowing tumor cells to evade the therapeutic effects. Additionally, mutations or overexpression of DNA repair-related genes in tumor cells are also associated with the development of resistance [5, 6]. Drugs targeting DNA repair pathways have become a highly focused area of research in cancer treatment. Inhibiting DNA repair induced by chemotherapy drug-induced DNA damage with PARP inhibitors is considered a novel therapeutic strategy for improving the efficacy of cancer therapy [7]. Inhibiting DNA repair pathways or using DNA damage repair inhibitors can enhance the sensitivity of tumor cells to treatment and overcome resistance. Understanding the intricate interplay between DNA repair pathways and cancer drug resistance is crucial for the development of effective therapies.

DNA methylation is a critical epigenetic modification and plays a pivotal role in gene epigenetic regulation, cancer and chemotherapy drug resistance. The alteration of DNA methylation has been found to be associated with the resistance of breast cancer cells to docetaxel [8]. The upregulation of Achete scutellike 2 (DNA transcription activator) through promoter demethylation, which has been shown to promote gastric cancer cell growth and confer resistance to 5-fluorouracil (5-FU) [9]. TET family that includes TET1, TET2 and TET3, are DNA enzymes which have been demonstrated to oxidize 5mC to 5hmC, 5fC, 5caC, and further to unmodified cytosine [10]. Aberrant DNA methylation is commonly associated with chemotherapy resistance in cancer [11]. In addition to its role as a demethylase, TET1 exhibits dual functions as a transcriptional activator and repressor, and its transcriptional activity may or may not depend on its demethylation activity [12]. TET1 has emerged as a multifaceted player involved in various processes related to cancer progression, including tumorigenesis and chemotherapy resistance. For instance, in 5-FU-resistant colorectal cancer cells, TET1 has been observed to be upregulated, and its involvement in active DNA demethylation has been linked to the acquisition of 5-FU resistance [13]. However, the precise mechanism by which TET1 regulates sorafenib resistance in hepatocellular carcinoma (HCC) remains to be elucidated.

The interaction between transcriptional regulators and epigenetic modifiers plays a crucial role in modulating gene expression and cellular processes. In recent years, extensive research has focused on understanding the crosstalk between these regulatory molecules and their implications in various biological contexts [14]. YAP1 has emerged as a key oncogene that play a critical role in various aspects of tumorigenesis and cancer progression [15]. In cancer cells, YAP1 is frequently overexpressed or hyperactivated and translocated into the nucleus, resulting in the transcriptional activation of target genes involved in cell proliferation, survival, and epithelial-mesenchymal transition (EMT) [16]. Beyond its involvement in tumorigenesis and cancer progression, YAP1 has been recognized as a significant contributor to therapeutic resistance in cancer [17]. Elevated activation of YAP1 expression is associated with resistance to chemotherapy and targeted therapies, impeding treatment efficacy [18]. Understanding the mechanistic basis and functional consequences of the YAP1-TET1 interaction is of significant interest due to its potential implications in cellular processes, disease pathogenesis, and therapeutic interventions.

In this study, we expounded the mechanism of sorafenib resistance mediated by TET1. TET1 contributed to sorafenib resistance of HCC cells by upregulating DNA repair-related gene through demethylation modification. Furthermore, TET1 inhibitor Bobcat339 can overcome sorafenib resistance and its combination with sorafenib had a significant therapeutic effect on HCC.

## Materials and methods

### Xenografts and histological analysis

Male BALB/c nude mice of 5 weeks old were subcutaneously injected in the upper flank with 5 × 10^6^ sorafenib-resistant Huh-7 cells (eight mice per group). After tumor establishment, mice were treated with DMSO, Bobcat339 alone (2.5 mg/kg/2 days, intraperitoneally), sorafenib alone (30 mg/kg/2 days, orally), or sorafenib + Bobcat339. Tumor volumes were calculated: volume (mm3) = L×W^2^ × 0.5; L: the largest diameters, W: the smallest diameters. Tumor volume was monitored every 2 days. For mst1^fl.^ mst2^fl.^ liver tumor mice, 4-month-old mice have already formed tumors and were randomly divided into four groups, with six mice in each group. Mice were treated with DMSO, Bobcat339, sorafenib or sorafenib + Bobcat339. After 3 weeks, tumor tissues were excised and fixed in formalin, then performed immunohistochemistry of anti-TET1 (1:150 dilution; GeneTex, catalog no. 124,207), anti-YAP1 (1:200 dilution; ABclonal, catalog no. 1002), or anti-γH2AX (1:150 dilution; Cell Signaling Technology, catalog no. 9718) antibody according to routine procedures. For tissue microarrays, perform immunostaining of TET1 and YAP1 protein, then interpreted immunohistochemical results by three professionals, finally, conduct statistical analysis of this interpretation results.

### CHIP-seq

For ChIP-seq experiments, sorafenib-resistant Huh-7 cells were fixed with formaldehyde followed by immediate termination with glycine. Subsequently, cells were washed and then lysed with SDS lysis buffer. Chromatin was sheared using an ultrasonic cell crusher (20% Hz, 15 cycles of 3 s on and 7 s off) on ice. Sheared chromatin of 200–300 bp was incubated with YAP1 antibody (catalog no. 1002; ABclonal) at 4 °C overnight. The fragmented DNA was eluted via the ChIP Assay Kit (Beyotime Biotechnology). DNA of immunoprecipitated chromatin and input chromatin were purified. Library construction and analysis were performed by Novogene Technology.

For the verification of ChIP–qPCR, the purified DNA was performed real-time PCR using specific primers for further analysis. The sequences of DNA primers for ChIP–qPCR are listed in the Supplementary Table S1.

### Human cell lines

The human HCC cell lines HepG2 and Huh-7 were provided by the Qiao Wu lab of Xiamen University. Sorafenib-resistant cell lines, sorafenib-resistant HepG2 cells and sorafenib-resistant Huh-7 cells were established from maternal HepG2 and Huh-7 cells by increasing concentrations of sorafenib treatment in a stepwise manner. These HCC cell lines were cultured in DMEM containing 10% serum and incubated at 37 °C with 5% CO2.

### Cell proliferation, cell apoptosis, and colony formation assay

HCC cells proliferation were counted by a Cell Counting Kit-8 assay. For apoptosis detection, cells were seeded into a six-well plate in triplicate and treated with reagents, flow cytometry analysis was conducted using the Annexin V-FITC Apoptosis Detection Kit (#BMS500FI-100, Invitrogen). For the colony formation assay, cells were cultured for 10 days with drugs treatment. Subsequently, crystal violet staining was performed.

### Real-time RT–PCR

RNA of HCC cells was extracted using TRIzol reagent and quantitated by the NANODROP LITE (ThermoFisher Scientific, Foster City, CA, USA). Subsequently, real-time PCR analysis was performed using specific primers. The forward and reverse primers are listed in the Supplementary Table S2. Besides, the shRNA sequence information are listed in the Supplementary Table S3.

### Extraction of cytoplasmic and nucleoprotein from cells

Mainly according to the instructions of the reagent kit (#FD0199, Fudebio-tech). Briefly, add 1 mL of reagent A to 1 × 10^7^ cells; After incubation on ice for 20 min, add 40 µL reagent B. Centrifuge at 4 degrees for 15 min, then collect the supernatant, which is the cytoplasmic protein. Add reagent N to the precipitate and then centrifuge for 15 min, collect the supernatant, which is the cell nucleoprotein.

### Western blotting

Cells were collected by PBS and lysed using RIPA lysis buffer, then the protein concentration was determined using the Bradford. Subsequently, SDS-PAGE was performed to separate the proteins, followed by incubation with specific primary and secondary antibodies. The bands were revealed using an ECL kit.

### Luciferase reporter assays

Briefly, transfection was performed using PEI according to the manufacturer’s instructions. Cells were collected after 48 h, then luciferase and β-galactosidase levels were measured. The fluorescence values of the control group were considered as 1 unit, and the fluorescence values of the treatment group were calculated relative to the control values.

### Alkaline comet assay

Mainly according to the instructions of the reagent kit (4250-050-K, R&D systems). Briefly, agarose gel was preheated to 37 °C until it became a liquid state. Then cells were immediately spread on the special slides. After the agarose solidified, it was immersed in the lysis solution. Subsequently, electrophoresis was performed in alkaline electrophoresis buffer at 21 V for 35 min. DNA staining was done using GoldView I, and the stained nuclei were visualized and captured under microscopy. Images were quantified using CASP software. The length of the comet tail was used as a parameter to assess the extent of DNA damage.

### Transcriptome sequencing

Transcriptome sequencing was performed in sorafenib-resistant Huh-7 cells treated with vehicle, 40 µM Bobcat339 and 80 µM Bobcat339. Library construction and analysis were performed by Novogene Technology.

### Human whole-genome methylation sequencing

The genome-wide methylation levels in control and shTET1 sorafenib-resistant Huh-7 cells were analyzed using 850 K BeadChip, which enables detection of the methylation levels of 853,307 CpG sites. Library construction and analysis were performed by Genesky Biotechnologies.

### Statistical analysis

Data were analyzed using GraphPad Prism software. Data are presented as the mean ± SD. All data were analyzed using two sided Student t tests, and *P* < 0.05 was considered statistically signifificant. **All authors had access to the study data and had reviewed and approved the final manuscript.**

## Results

### TET1 is upregulated in human HCC tissues and predicts a poor prognosis

**Fig. 1:**
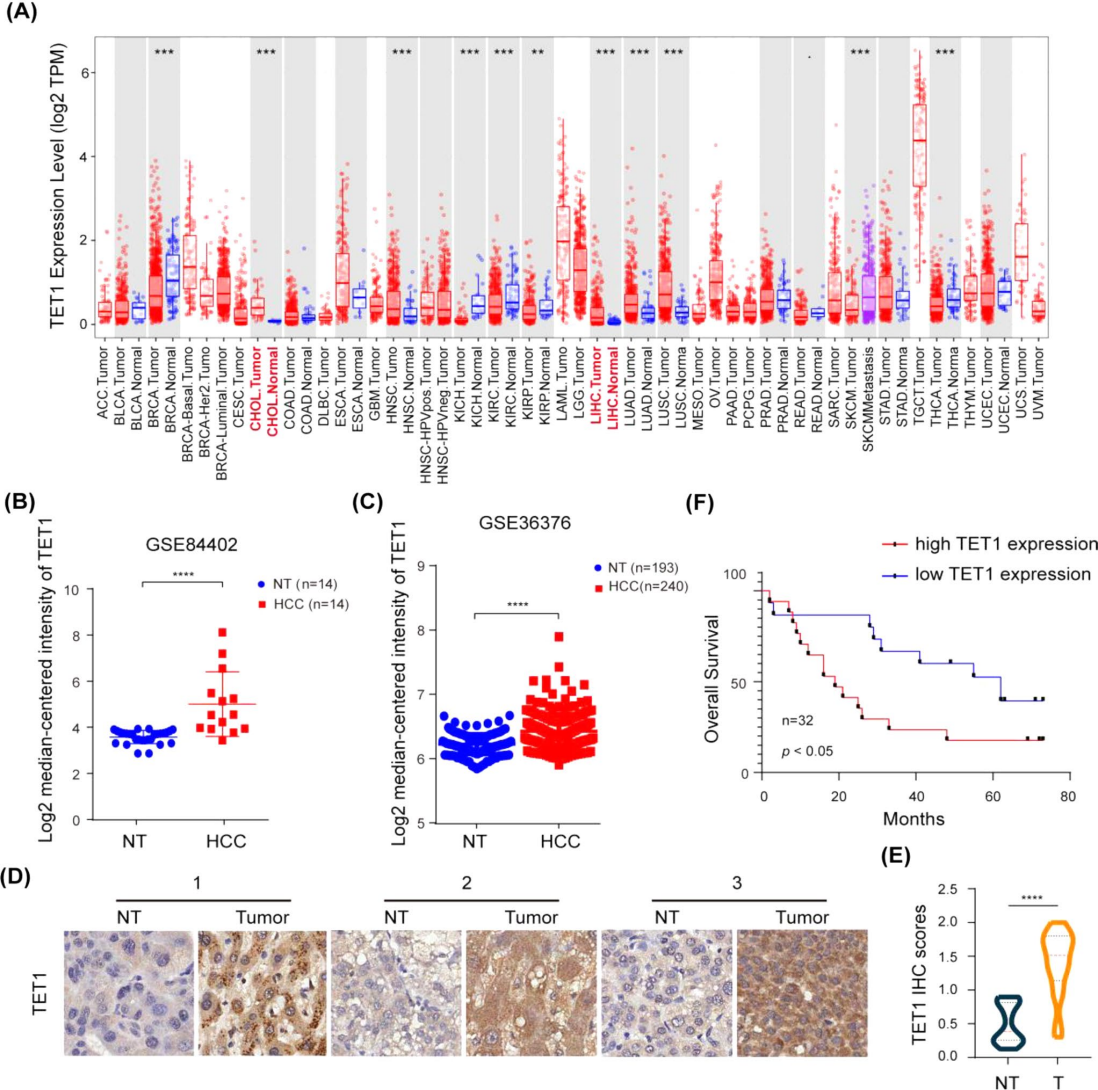
TET1 expression is upregulated in HCC tissues and predicts a poor clinical outcome. **(A)** TET1 expression among 19 types of cancer in RNA-seq data of TCGA project. **(B)** The mRNA level of TET1 in 14 pairs HCC tissues and corresponding non-cancerous tissues revealed using the GSE84402 datasets. **(C)** The mRNA level of TET1 in 240 liver tumors and 193 adjacent non-tumor livers revealed using the GSE36376 datasets. **(D-E)** Immunohistochemical staining of TET1 protein on 21 pairs HCC tumor tissues and corresponding non-cancerous tissues as indicated by the representative samples **(D)** and the summary of all the samples **(E)**. **(F)** Overall survival of HCC patients was assessed using Kaplan-Meier plots  (*P* values are shown in the graphs). Data represent the means ± SD. *P < 0.05; ****P < 0.0001. Abbreviation: NT, non-tumorous liver

To investigate the roles of TET1 in tumors, we first performed an analysis of TCGA database and found that TET1 mRNA level varies across different types of cancer. TET1 showed both very low expression in normal tissue and significant upregulation in tumor only in HCC and Cholangiocarcinoma (CHOL) (Fig. 1A). Therefore, we focus on HCC in this study. By analyzing on the GEPIA database, we had observed that the mRNA level of TET1 was higher in HCC compared to normal tissues (Fig. S1A). To further verify the above notion, we downloaded publicly available datasets GSE84402, GSE36376 and GSE6764, and performed data transformation analysis. The results revealed a significant upregulation of TET1 expression in HCC, comparing with both the paired (Fig. 1B) or non-paired (Fig. 1C and S1B) non-cancerous tissues. Immunohistochemical staining of TET1 protein was also performed on tissue microarrays with 21 cases of HCC tissues and corresponding non-cancerous tissues (Table 1). The results showed that the TET1 protein expression of HCC tissues was significantly higher than corresponding adjacent tissues as indicated by the representative samples in the tissue microarrays (Fig. 1D) and the summary of all the samples (Fig. 1E). Moreover, patients with high TET1 expression displayed a poor overall survival (Fig. 1F).

**Table 1:** The paired HCC patients characteristics

Characteristics	Number of cases	Percentages
Clinical stage		
Stage I	6	29%
Stage II	11	52%
Stage III	4	19%
OS		
Alive	7	33%
Dead	14	67%
Number of lesions		
0	2	10%
1	18	86%
2	1	5%
Metastasis		0%
YES	21	100%
NO	0	0%
Age		0%
<60	15	71%
≥ 60	6	29%

OS: overall survival

### Inhibition of TET1 sensitizes HCC cells to sorafenib treatment

**Fig. 2:**
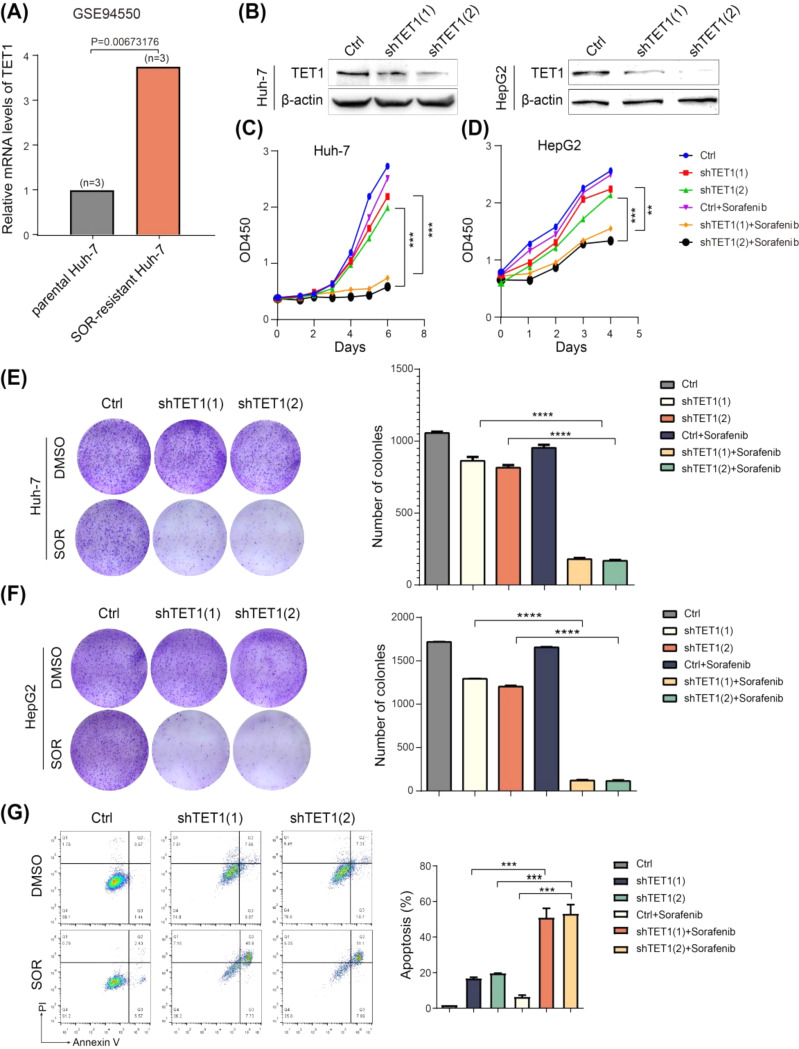
Inhibition of TET1 sensitized HCC cells to sorafenib treatment. **(A)** Analysis of TET1 expression was performed of GSE94550 dataset, which was the RNA-seq of parental and sorafenib-resistant Huh-7 cells (*P* value is shown in the graph). **(B)** Huh-7 (left) and HepG2 (right) cells were stably expressed with Ctrl shRNA or two different TET1 shRNAs. Protein levels of TET1 were detected by western blotting. **(C-D)** Cell proliferation (cell counting kit 8) of HCC cells posttransfection with Ctrl shRNA or TET1 shRNA upon sorafenib treatment. **(E-F)** Representative photos (left) and quantification (right) of colony formation assays. **(G)** Huh-7 cell apoptosis induced by sorafenib were detected by Flow cytometry with Annexin V/PI. Data are expressed as the means ± SD of three independent experiments. **P < 0.01; ***P < 0.001; ****P < 0.0001. Abbreviation: SOR, sorafenib

**Fig. 3:**
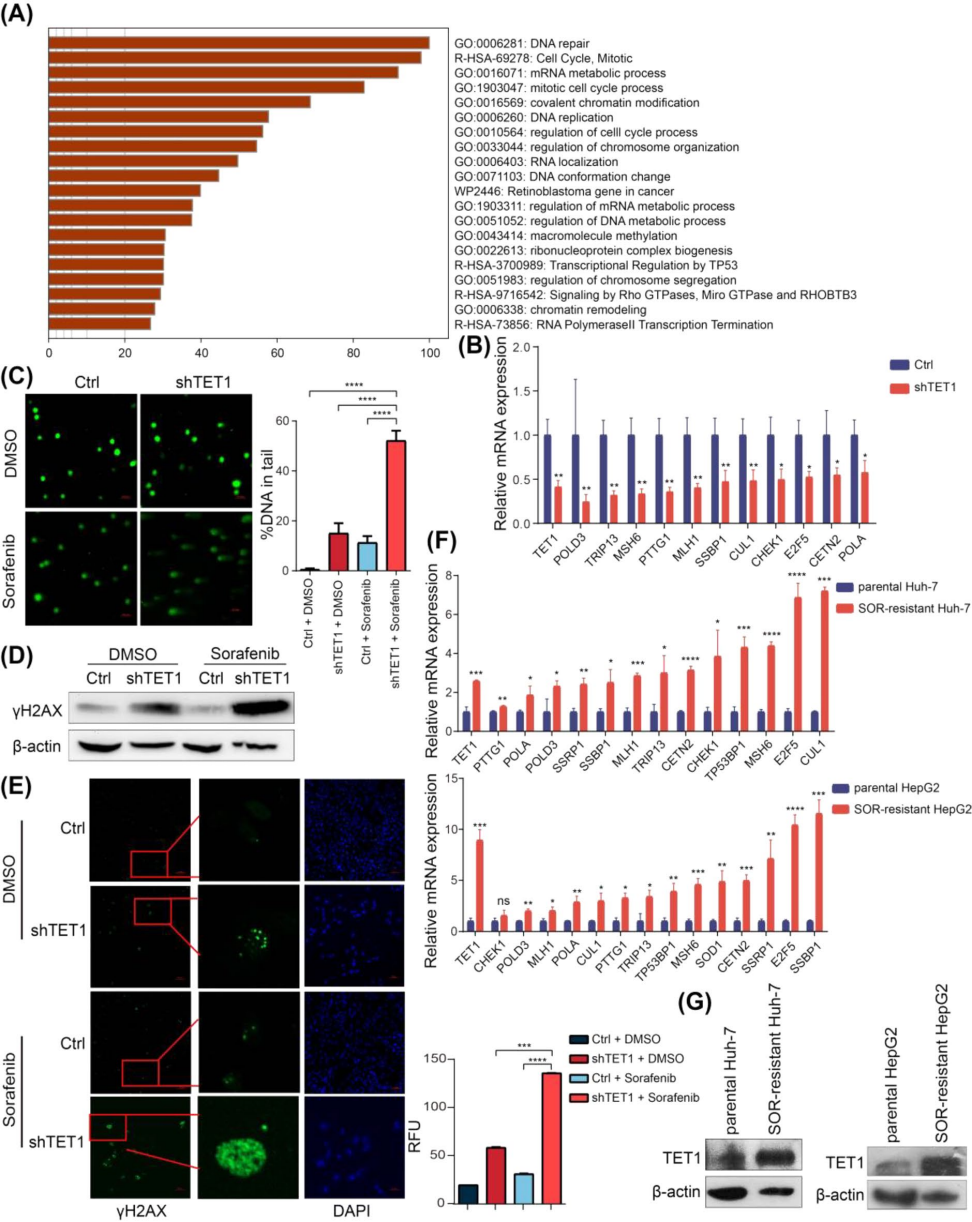
The DNA damage repair signaling upregulated by TET1 might be critical in sorafenib resistance. **(A)** DAVID gene ontology analysis of 3,667 genes which were positively related to TET1 in HCC. **(B)** The mRNA levels of DNA repair-related genes in shTET1 and control Huh-7 cells were measured by RT-qPCR. **(C)** Huh-7 cells with ctrl and shTET1 were subjected to treatment with a nontoxic dose of 2 µM sorafenib. DNA damage were measured by alkaline comet assays, representative comet tails were shown (left), the percentage of DNA in the comet tail were summarized from at least 50 cells (right), statistical analysis was conducted using GraphPad software. **(D)** γH2AX protein level in Huh-7 cells was measured by western blotting. **(E)** Immunofluorescence was performed to measured γH2AX expression and aggregation in the nucleus of Huh-7 cells. **(F)** The mRNA levels of TET1 and DNA repair-related genes in sorafenib-resistant HCC cells were measured by qPCR. **(G)** TET1 protein levels were increased in sorafenib-resistant HCC cells. Data are expressed as the means ± SD of three independent experiments. *P < 0.05; **P < 0.01; ***P < 0.001; ****P < 0.0001. Abbreviation: RFU, Relative Fluorescence Units

Analysis of the GSE94550 dataset showed that in sorafenib-resistant Huh-7 cells, TET1 mRNA expression is upregulated (Fig. 2A), while the expression levels of TET2 and TET3 remain unchanged (Fig S2A, B), which was verified in our sorafenib-resistant Huh-7 cells by RT-qPCR (Fig. 3F). This suggests that TET1 may play a certain role in the process of sorafenib resistance, while TET2 and TET3 may not play a critical role in this process. Then, we used shRNA-TET1 to specifically knock down TET1 expression in Huh-7 and HepG2 (Fig. 2B), and treated them with a nontoxic dose (2 µM) of sorafenib. The results showed that TET1 knockdown significantly increased the killing effect of sorafenib on tumor cells (Fig. 2C-F), although TET1 knockdown alone also inhibited the proliferation of HCC cells to a certain degree. Flow cytometry by Annexin V/PI staining showed that TET1 knockdown also significantly promoted sorafenib-induced apoptosis (Fig. 2G). These results indicate that inhibition of TET1 significantly enhances the sensitivity of HCC cells to sorafenib.

### TET1 affects the sensitivity of HCC cells to sorafenib by upregulating DNA repair genes

To explore the downstream molecular mechanism by which TET1 affects sorafenib sensitivity in HCC cells, we obtained a list of 3,667 genes that are positively related to TET1 in HCC from the UALCAN database. We then performed GO and KEGG enrichment analyses based on these genes. Pathway analysis indicated that these genes mainly enriched in DNA repair pathway (Fig. 3A). In addition, we wondered whether TET1 regulates the expression of these genes in HCC cells. RT-PCR results showed that DNA repair-related genes were downregulated in shTET1 Huh-7 cells (Fig. 3B). Next, 2 µM sorafenib was used to treat shTET1 and control HCC cells, and alkaline comet assay showed that TET1 knockdown significantly increased the length of DNA tails indicating that DNA damage was more severe in shTET1 Huh-7 cells (Fig. 3C) and HepG2 cells (Fig. S3A). Phosphorylation of histone H2AX (γH2AX) is another marker of DNA damage, reflecting the production and repair of DNA damage. Immunoblotting results revealed that TET1 knockdown significantly induced the expression of γH2AX protein (Figs. 3D and S3B). The immunofluorescence results showed that TET1 knockdown significantly increased the expression and aggregation of γH2AX in the nucleus (Fig. 3E). These results suggest that TET1 knockdown reduced the expression of DNA repair-related genes, resulting in more severe DNA damage caused by nonlethal doses of sorafenib. Next, we produced sorafenib-resistant Huh-7 and HepG2 cell lines. The decreased number of apoptotic cells under high-dose (10 µM) sorafenib treatment proved the successful construction of sorafenib-resistant cells (Fig. S4A, B). Then, 10 µM sorafenib was used as the maintenance dose of resistant cells. Western blotting results demonstrated that the TET1 protein level was increased in sorafenib-resistant cells (Fig. 3G), while there are no significant changes in TET2 and TET3 (Fig. S4 C). In addition, the mRNA level of TET1 and DNA repair-related genes were significantly increased in the sorafenib-resistant cells (Fig. 3F).

### TET1 is upregulated by YAP1 signaling in HCC cells

**Fig. 4:**
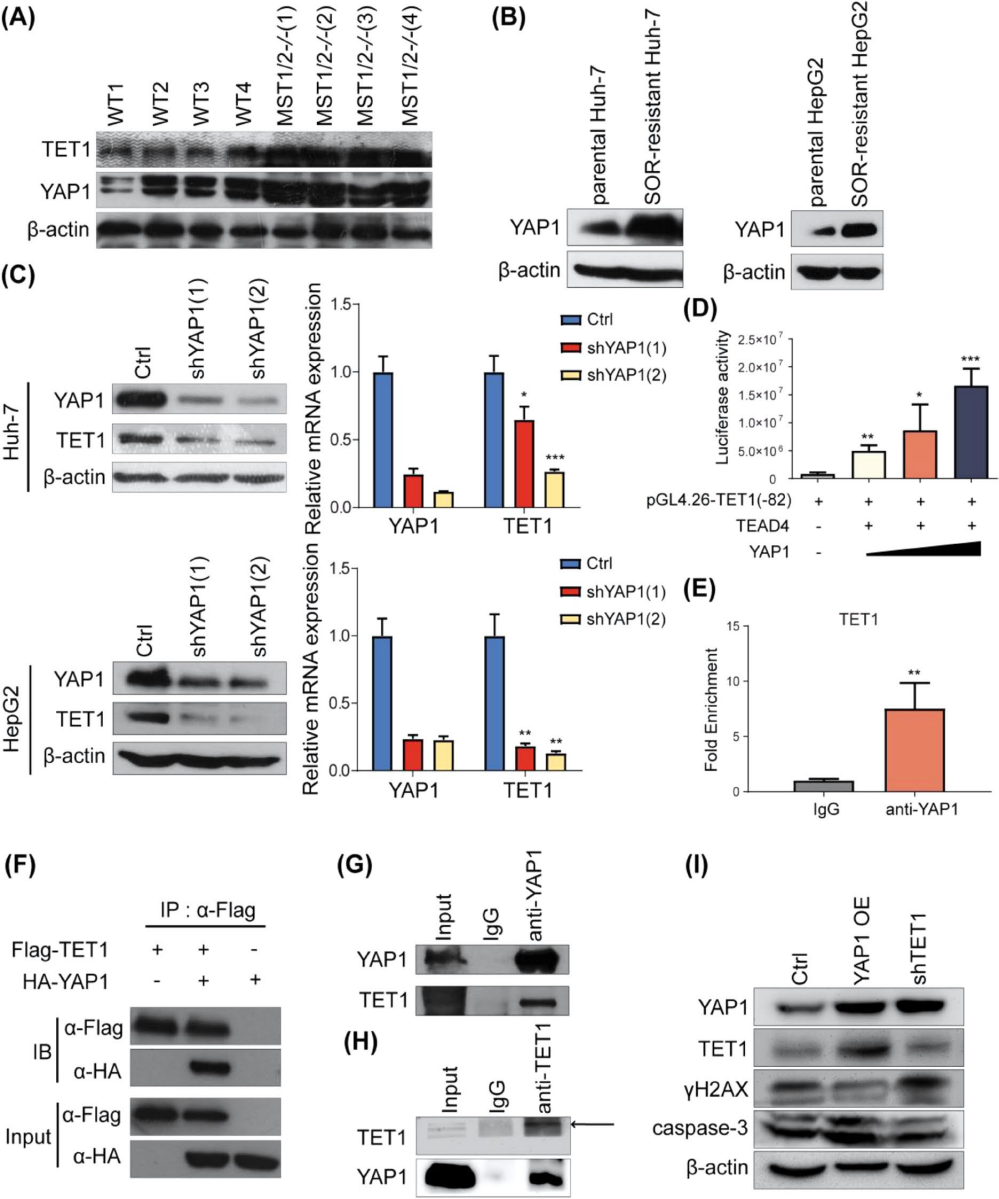
TET1 is upregulated by YAP1 signaling in HCC cells. **(A)** Analysis of the correlation between TET1 and YAP1 in the same HCC tissues, indicated by the representative samples (left) and the summary of all the samples (right). Scale bars = 50 μm. **(B)** YAP1 protein was upregulated in sorafenib-resistant HCC cells compared to parental cells. (C) When YAP1 was knocked down in sorafenib-resistant HCC cells, TET1 protein (left) and mRNA (right) levels were measured by western blotting and qPCR analysis. **(D)** Luciferase reporter assays in sorafenib-resistant Huh-7 cells demonstrated a dose-dependent increase in the activity of the TET1 luciiferase reporter upon YAP1 overexpression. **(E)** ChIP experiments in sorafenib-resistant Huh-7 cells indicated that YAP1 binds to the TET1 promoter. **(F)** Sorafenib-resistant Huh-7 cells were co-transfected with HA-YAP1 and Flag-TET1, and the results of Co-IP assays demonstrated the association between TET1 and YAP1. **(G)** and **(H)** Co-IP experiments were conducted to detect the interaction between endogenous TET1 and YAP1 in sorafenib-resistant Huh-7 cells. **(I)** Rescue experiments in sorafenib-resistant Huh-7 cells indicated that YAP1 contributes to sorafenib resistance of HCC cells by upregulating TET1 expression. Data are expressed as the means ± SD of three independent experiments. *P < 0.05; **P < 0.01; ***P < 0.001. Abbreviation: WT, wild type; IB, immunoblotting; IgG, immunoglobulin G

What is the molecular mechanism underlying the upregulation of TET1 in sorafenib-resistant HCC cells? The mechanism was first investigated in our mst1^fl.^ mst2^fl.^ liver tumor mouse model. In this model, liver tumor mainly induced by YAP1 overexpression caused by inhibition of hippo signaling pathway. YAP1 is a downstream effector of the hippo signaling pathway and associated with cancer. Western blotting showed that TET1 protein expression increased along with YAP1 expression in mst1/2 mutant mice tumor tissues (Fig S5A), which suggests that TET1 expression may be regulated by YAP1 in HCC cells. We investigated the relationship between TET1 and YAP1 protein expression in the same set of HCC samples (Table 2) and observed a positive correlation between their levels (Fig. 4A). Then we examined YAP1 protein expression in sorafenib-resistant HCC cells and immunoblotting results revealed a significant increase in the protein expression of YAP1 in sorafenib-resistant HCC cells (Fig S5B). When YAP1 was knocked down in sorafenib-resistant Huh-7 and HepG2 cells, TET1 protein and mRNA levels were significantly reduced (Fig. 4B). Since YAP1 usually forms a transcription complex with TEAD and TEAD is the DNA binding component of the YAP1/TEAD transcription complex, we then checked the TET1 promoter region and found a TEAD binding sequence conserved between mice and humans at the − 82 site. Luciferase reporter assays indicated dose-dependent transcriptional activation of TET1 by YAP1 (Fig. 4C). This result was further confirmed by using a chromatin immunoprecipitation (ChIP) assay (Fig. 4D). Since TET1 is usually recruited to the specific gene promoter by interacting with transcription factors to play its role, we asked whether TET1 and YAP1 interact with each other. The co-immunoprecipitation (Co-IP) assays in Flag-TET1 and HA-YAP1 transfected sorafenib-resistant Huh-7 cells indicated that TET1 associated with YAP1 (Fig. 4E). Finally, we performed immunoprecipitation (IP) assay of nuclear extracts from sorafenib-resistant Huh-7 cells. Endogenous IP of YAP1 pulled down TET1 (Fig. 4F). Accordingly, endogenous IP of TET1 pulled down YAP1 (Fig. 4G). The protein subcellular location is important to its function. It was found on the website (https://www.genecards.org/) that YAP1 protein is evenly distributed in the cytoplasm and nucleus (Fig S5C), and TET1 protein is slightly higher in the nucleus than in the cytoplasm (Fig S5D). In addition, we conducted nuclear plasma separation experiments and found that YAP1 and TET1 proteins in the nuclei of sorafenib- resistant HCC cells were significantly higher than those in the cytoplasm. Additionally, the expression of YAP1 and TET1 protein were significantly higher in the resistant cells, both in the nucleus and cytoplasm. These indicate that prolonged exposure to sorafenib can significantly induce more YAP1 protein aggregation in the nucleus, thus inducing the up-regulated expression of TET1 and DNA repair-related genes (Fig S5E). We also conducted a rescue experiment. Western blotting showed that upon sorafenib treatment, YAP1 overexpression in the sorafenib-resistant Huh-7 cells resulted in less γH2AX and more caspase-3 protein expression; Overexpressing YAP1 while simultaneously knocking down TET1 increased the γH2AX and reduced the caspase-3 protein expression (Fig. 4H). These results suggest that TET1 is upregulated by YAP1 and interacts with YAP1 to promote drug resistance-related gene expression.

**Table 2:** The clinical characteristics of 32 HCC patients

Characteristics	Number of cases	Percentages
Clinical stage		
Stage I	7	22%
Stage II	18	56%
Stage III	7	22%
OS		
Alive	10	31%
Dead	22	69%
Number of lesions		
0	3	9%
1	27	84%
2	2	6%
Metastasis		
YES	32	100%
NO	0	0%
Age		
<60	25	78%
≥ 60	7	22%

OS: overall survival

### TET1 and YAP1 synergistically regulate the promoter methylation and gene expression of DNA repair-related genes

**Fig. 5:**
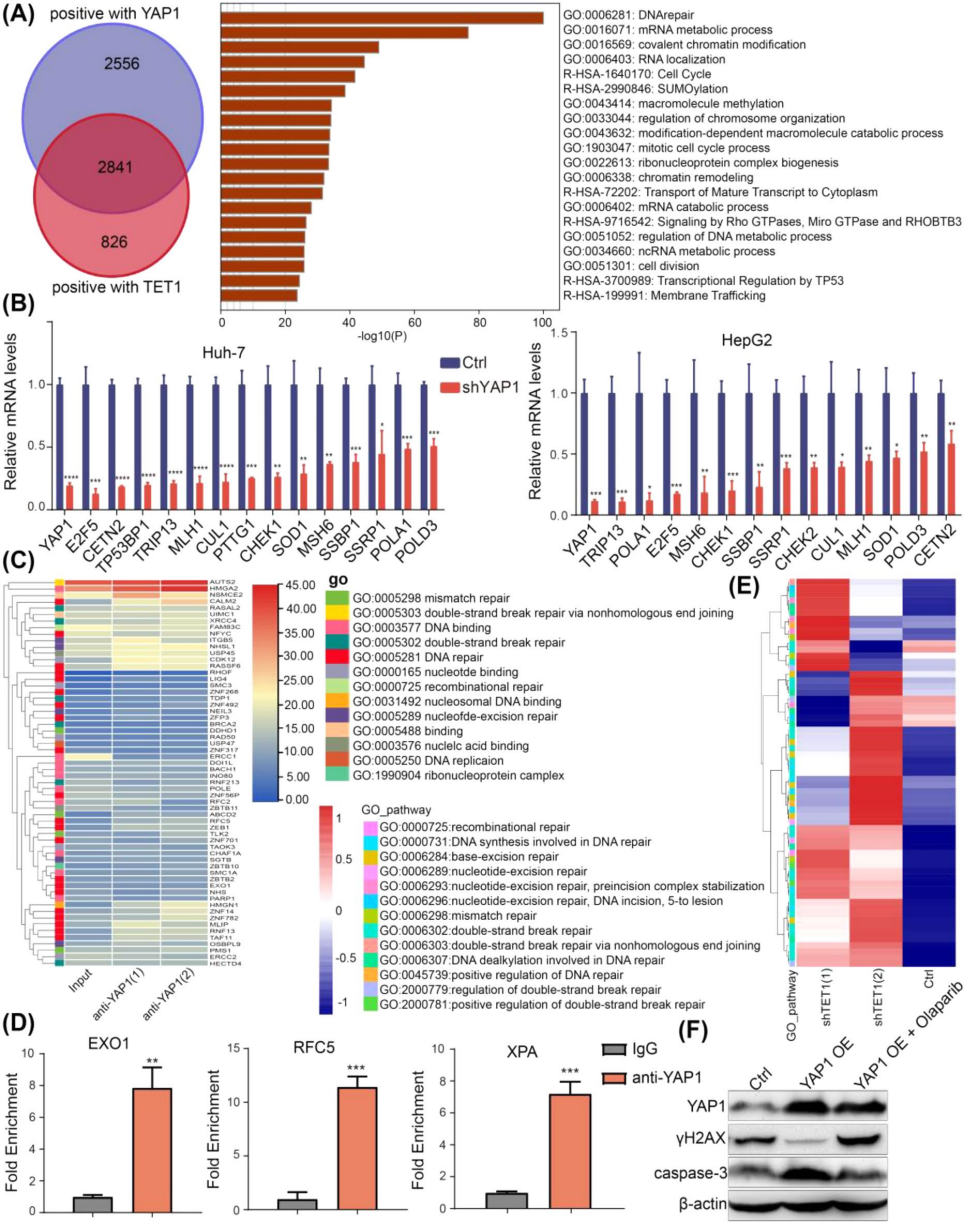
TET1 and YAP1 synergistically regulate the promoter DNA methylation and gene expression of DNA repair-related genes. **(A)** A list of 5,397 genes positively related to YAP1 in HCC was obtained from the UALCAN database, and genes in the intersection of these two datasets were mainly enriched in the DNA repair pathway. **(B)** RT**-**qPCR analysis of DNA repair-related gene expression in ctrl and shYAP1 sorafenib-resistant Huh-7 and HepG2 cells. **(C)** YAP1-specific ChIP-seq in sorafenib-resistant Huh-7 cells showed that YAP1 binds to the promoters of DNA repair-related genes. **(D)** ChIP–qPCR analysis in sorafenib-resistant Huh-7 cells of the DNA repair genes EXO1, XPA and RFC5. **(E)** Whole-genome bisulfite sequencing of Ctrl and TET1 knockdown sorafenib-resistant Huh-7 cells. **(F)** Rescue experiments in sorafenib-resistant Huh-7 cells demonstrated that TET1 which upregulated by YAP1 contributes to sorafenib resistance in HCC cells, primarily through the activation of DNA damage repair signaling pathways. Data are expressed as the means ± SD of three independent experiments. *P < 0.05; **P < 0.01; ***P < 0.001; ****P < 0.0001

We investigated signaling pathways coregulated by TET1 and YAP1. We obtained a list of 5,397 genes positively associated with YAP1 and a list of 3,667 genes positively associated with TET1 in HCC from the UALCAN database. Interestingly, genes in the intersection of these two datasets were mainly enriched in the DNA repair pathway (Fig. 5A). DNA repair-related genes were downregulated when YAP1 was knocked down in sorafenib-resistant Huh-7 and HepG2 cells (Fig. 5B). Since YAP1 is a transcriptional co-activator and TEAD4 is the main binding partner [19], we wondered whether YAP1 is recruited to the promoter of DNA repair-related genes. The YAP1-specific ChIP-seq results revealed that YAP1 indeed binds to the promoter of these genes (Fig. 5C). This result was validated by ChIP–qPCR of selected genes: EXO1 [20], XPA [21] and RFC5 [22], which are implicated in different stages of DNA damage repair process. (Fig. 5D). TET1 is a demethylase that regulates gene expression by altering DNA methylation status. Whole-genome bisulfite sequencing revealed that the DNA methylation levels of DNA repair-related genes were upregulated in shTET1 sorafenib-resistant Huh-7 cells (Fig. 5E). Besides, we searched the UCSC genome browser and found there are YAP1/TEAD4 binding sites on the enhancer/promoter of the most of these DNA repair-related genes, and these binding sites are located near CpG islands (Fig S6), which further validates our conclusion. We also conducted a rescue experiment. Western blotting showed that upon sorafenib treatment, YAP1 overexpression in the sorafenib-resistant Huh-7 cells resulted in less γH2AX and more caspase-3 protein expression; treating YAP1-overexpressed sorafenib-resistant Huh-7 cells with olaparib (inhibitor of DNA damage repair) increased the γH2AX and inhibited the caspase-3 protein expression (Fig. 5F). The above results suggest that the YAP1 protein level is increased in sorafenib-resistant HCC cells. Consequently, YAP1 promote TET1 expression and recruited TET1 to the promoters of DNA repair-related genes, resulting in the demethylation and expression of these genes.

### TET1 inhibitor can reverse sorafenib resistance in HCC cells

**Fig. 6:**
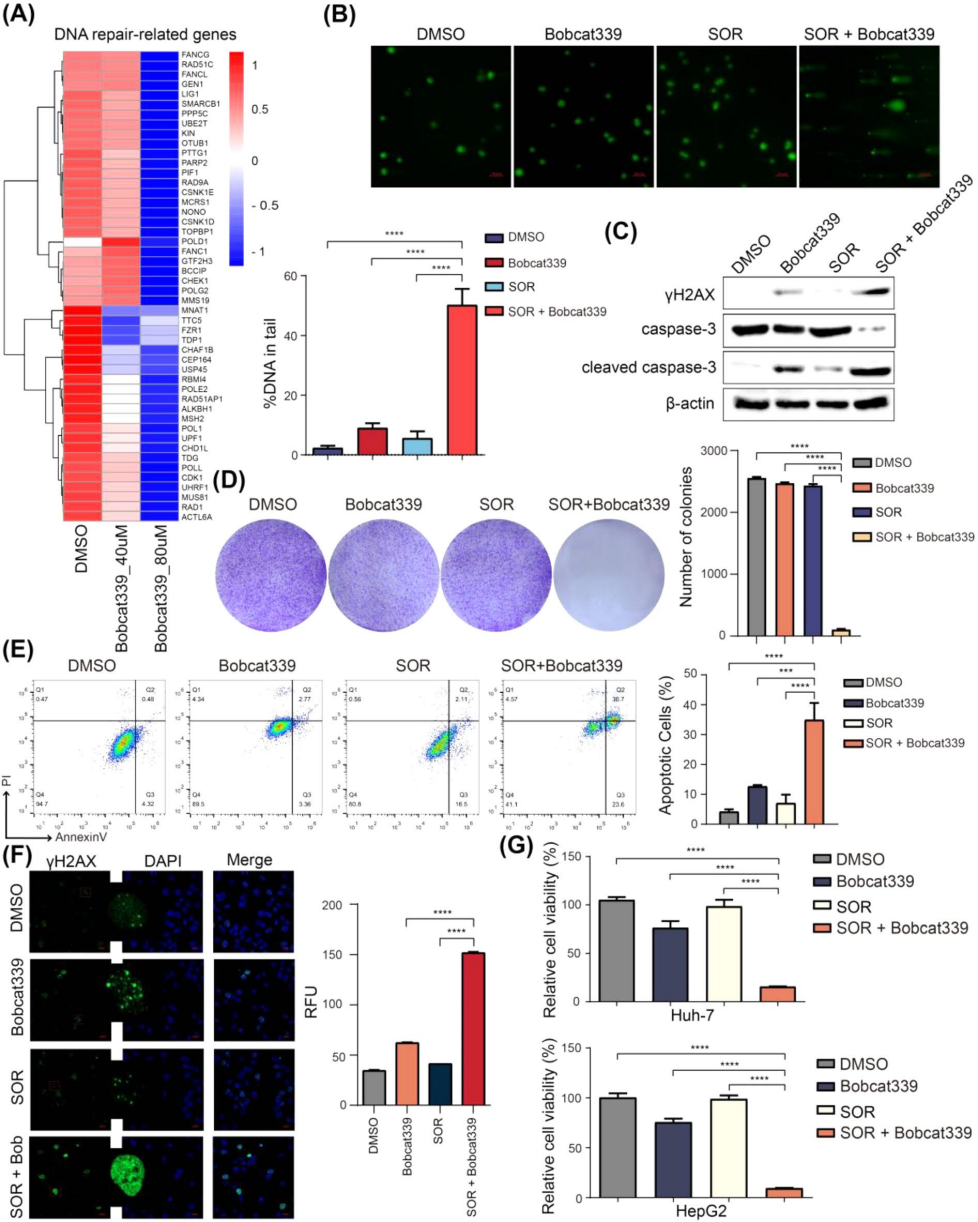
TET1 inhibitor treatment reverses sorafenib resistance of HCC cells. **(A)** RNA-seq of sorafenib-resistant Huh-7 cells with 40 µM and 80 µM Bobcat339 treatment. **(B-G)** Sorafenib-resistant Huh-7 cells treated with DMSO, 10 µM sorafenib alone, 40 µM Bobcat339 alone, 10 µM sorafenib and 40 µM Bobcat339 together. DNA damage of the four groups were measured by the comet assays**(B)**; The protein levels of the apoptosis markers caspase-3 and cleaved caspase-3, and the DNA damage marker γH2AX were measured by western blotting**(C)**; Cell proliferation was assessed using colony formation assay**(D)**; The cell apoptosis of the four groups were evaluated using flow cytometry analysis **(E)**; The expression and aggregation of γH2AX in the nucleus of sorafenib-resistant Huh-7 cells were detected by Immunofluorescence experiments, Scale bar, 20 μm**(F)**; Cell growth of HCC cells measured by CCK-8 assay**(G)**. Data are expressed as the means ± SD of three independent experiments. ***P < 0.001; ****P < 0.0001

**Fig. 7:**
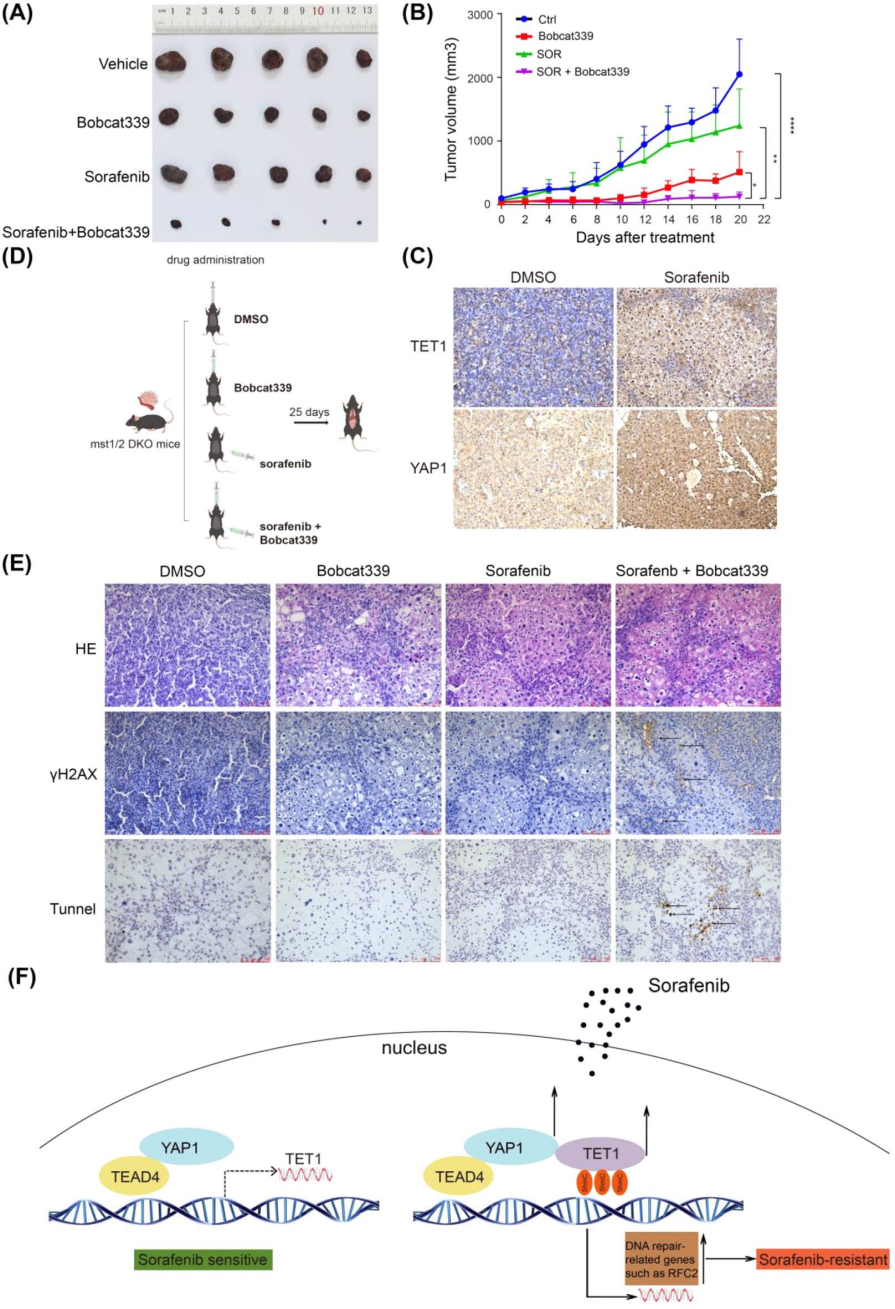
TET1 inhibitor reverses sorafenib resistance and TET1 correlates positively with YAP1 in HCC in vivo. **(A-B)** Nude mice were subcutaneously injected with sorafenib-resistant Huh-7 cells and randomly divided into four groups, treated with sorafenib alone, Bobcat339 alone, sorafenib and Bobcat339, or vehicle controls, comparison tumor size **(A)** and tumor growth **(B)** among the four mice groups. Error bars represent the means ± SD from *n* = 5 mice per group. *P < 0.05; **P < 0.01; ***P < 0.001; ****P < 0.0001. **(C)** The protein expression levels of TET1 and YAP1 were increased in subcutaneous tumors from the sorafenib alone group compared with the vehicle group. Scale bar, 75 μm. **(D**) Illustration of drug administration in liver tumor mice. This image was drawn by Figdraw. (**E)** Immunohistochemistry and TUNEL staining were performed on tumor tissues. Scale bar, 100 μm. **(F)** A proposed model illustrating the working model of the upstream and downstream mechanisms of sorafenib resistance induced by TET1 in HCC cells

Bobcat339 hydrochloride is a potent and selective cytosine-based TET1 enzyme inhibitor. Treatment of sorafenib-resistant Huh-7 cells with Bobcat339 significantly reduced the expression of DNA repair-related genes (Fig. 6A). To assess whether the TET1 inhibitor can reverse sorafenib resistance, we treated sorafenib-resistant Huh-7 cells with 10 µM sorafenib (maintenance dose) alone, 40 µM Bobcat339 alone, combination of sorafenib and Bobcat339, and vehicle controls for 48 h. The comet assay showed that combined drug treatment resulted in more DNA damage (Figs. 7B and S7A). Western blotting showed that the apoptotic markers and the DNA damage maker were not significantly changed in the sorafenib alone group, but these makers were changed slightly when treated with Bobcat339 alone compared with control group. However, the expression of γH2AX and cleaved caspase-3 increased, whereas full caspase-3 significantly reduced in combination group, indicating that there was more DNA damage and apoptosis in the cells with combination therapy (Figs. 6C and S7B). The colony formation assay showed a marked decrease in both the colony formation ratio and colony size in combination group (Fig. 6D). And cell apoptosis was increased in combination group (Figs. 6E and S7C). In addition, the immunofluorescence results showed that the combination therapy significantly increased the expression and aggregation of γH2AX in the nucleus, indicating more DNA damage in the cells (Fig. 6F). Moreover, CCK-8 assays showed that combination therapy significantly inhibited cell proliferation (Fig. 6G). These results demonstrated that TET1 inhibitor could overcome sorafenib resistance and its combination with sorafenib exhibited significant therapeutic efficacy against HCC cells.

### TET1 inhibitor reverses sorafenib resistance in HCC in vivo

Finally, we performed in vivo experiments. Nude mice were subcutaneously injected with sorafenib-resistant Huh-7 cells. Sorafenib alone did not significantly reduce HCC growth in vivo, while combined treatment almost completely halted HCC growth, although Bobcat339 alone only slightly reduced HCC growth (Fig. 7A, B and Fig S8A, B). Resected xenografts were harvested for mechanistic analyses to test whether antitumor activity of Bobcat339 correlated with alterations in DNA damage repair events. Combined treatment group resulted in a marked increase in γH2AX immunostaining, and an increase in apoptotic cells as observed through TUNEL staining (Fig S8C). TET1 and YAP1 protein levels were increased in subcutaneous tumors from sorafenib alone group compared to vehicle group, indicating that long-term administration of sorafenib induces the expression of TET1 and YAP1, which is consistent with the results obtained from in vitro experiments (Fig. 7C). Besides, we performed the drug administration with mst1/2 mutant mice (Fig. 7D). Immunohistochemical and immunoblotting result indicated combined treatment resulted in a marked increase in γH2AX protein level (Fig. 7E and Fig S8D). And tunnel staining showed that combined treatment induced more apoptosis cells (Fig. 7E). In summary, our study uncovers a crucial regulatory mechanism underlying sorafenib resistance involving TET1 (Fig. 7F).

## Discussion

The development of drug resistance remains a significant challenge in the therapeutic efficacy of sorafenib. In order to improve the susceptibility of cancer cells to anticancer drugs, it is crucial to gain a deeper understanding of the underlying mechanisms driving sorafenib resistance and to develop effective strategies to counteract it. Therefore, investigating the mechanisms underlying the acquisition of sorafenib resistance was important. Currently, there is a lack of reliable and robust biomarkers that can accurately predict the sensitivity of HCC to sorafenib. Therefore, it is valuable to explore the utilization of pretreatment biomarkers as guidance for sorafenib treatment. In our studies, we have identified a novel role of TET1 in promoting DNA repair processes during the development of sorafenib resistance in HCC. These findings expound the potential involvement of TET1 in the resistance mechanisms and offer new insights into the development of effective therapeutic approaches to overcome sorafenib resistance in HCC.

Numerous studies have provided evidence highlighting the significance of TET1 in conferring drug resistance in various types of cancer [23–25]. DNA methylation is an important epigenetic modification in gene expression and is involved in the regulation of many biological processes [26]. Previous studies suggest that one of the mechanisms of drug resistance of tumor cells to chemotherapy is the change in the DNA methylation pattern affecting gene expression [11, 27, 28]. Our experiments revealed that TET1 was recruited to the promoter of DNA repair-related genes and activated gene expression through promoter demethylation, which leaded to sorafenib resistance in HCC cells. Bobcat339 sensitized and enhanced the therapeutic efficacy of sorafenib, offering a promising approach to augment the antitumor effects of sorafenib. Here, in the xenografts model, Bobcat339 alone group showed a certain inhibitory effect of tumor growth. It is important to note that as the experiment approached the ethically permissible duration, the combination therapy demonstrated minimal additional tumor growth and exhibited no increasing trend. Conversely, when treated with Bobcat339 alone, the tumor continued to grow, albeit at a slower rate. The rapid proliferation of tumor cells is well-documented, resulting in a high frequency of replication errors that necessitate repair mechanisms. Bobcat339 alone did not induce DNA damage, while combination therapy induced a large amount of DNA damage and apoptosis (Fig S8C). In our study, Bobcat339 inhibits the repair process, thereby decelerating the proliferation rate of tumor cells. Nevertheless, it does not exhibit significant cytotoxic effects. Upon discontinuation of treatment, tumor cells readily resume rapid proliferation due to the release of repair inhibition. For eradication of tumor cells, the use of cytotoxic chemotherapy drugs such as sorafenib is necessary. Furthermore, combined therapy may decrease potential unwanted sorfenib toxicity by lowering its effective dosage and can avoid unnecessarily suffering from the serious and sometimes lasting side effects of chemotherapy, including increased risk of secondary primary cancer.

YAP1 is protective and helps maintain the survival of malignant cells under chemotherapy drug treatment [29, 30]. In the present study, we demonstrated that YAP1 can promote TET1 activation, resulting in reduced sorafenib sensitivity in HCC cells. It has been reported that there are mainly two transcripts (TET1-FL and TET1-short) of TET1 in a variety of tumor cells [31, 32]. Our results show that both transcripts are expressed in HCC cells (Fig S9A), but only TET1-FL is regulated by YAP1 (Fig S9B). YAP1 interacts with TET1 to promote DNA repair-related gene expression, leading to sorafenib resistance in HCC cells. A positive relationship between tumor YAP1 and TET1 levels was then validated in MST1/2^*−/−*^ mouse tumor tissues (Fig S5A) and clinical HCC tissues (Fig. 4A). However, why YAP1 is highly expressed in sorafenib-resistant HCC cells is still unclear. We speculate that this might be due to the following two reasons. First, since sorafenib is a multikinase inhibitor, it is possible that sorafenib directly inhibits the activity of upstream kinases of YAP1, such as MST1/2 and LATS1/2, resulting in upregulation of YAP1 protein levels. Second, some studies indicate that intratumor heterogeneity complicates treatment failure, and intratumor heterogeneity is a common in tumor tissues. Therefore, after long-term treatment with sorafenib, liver cancer cells with low YAP1 expression are eliminated, leaving cells with high YAP1 expression, resulting in increased YAP1 expression in sorafenib-resistant HCC cells. The specific mechanism of YAP1 upregulation in sorafenib-resistant HCC cells is worthy of further study.

Many chemotherapeutic drugs induce DNA damage, thereby activating DNA repair of cells. In HCC, overexpression of DNA damage repair genes has been observed, leading to resistance against sorafenib treatment [33]. Similarly, DNA repair has been implicated in conferring resistance to cisplatin in human testis tumor cells [34] and epirubicin in breast cancer [35]. These findings, combined with our own results, provide further evidence supporting the significance of DNA repair in the adaptive survival response during drug treatment. Our results showed that TET1 can change the methylation status of DNA repair-related genes in sorafenib-resistant HCC cells. Among the genes affected by TET1-mediated changes in methylation status, several notable ones include HMGN1, NSMCE2, UIMC1, RFC2 and HMGA2, most affect the proliferation of HCC cells under sorafenib treatment (Fig S10). Given these findings, it is conceivable that monitoring the methylation levels of these DNA repair-related genes in the blood could potentially establish a method for monitoring drug resistance. This approach holds promise for predicting the development of drug resistance in patients and guiding the implementation of alternative treatment strategies.

In conclusion, our study identified a complex biologic network linking TET1 to the modulation of sorafenib resistance in HCC. Our findings have provided evidence that TET1 promotes DNA damage repair in sorafenib-resistant HCC cells. Importantly, we have demonstrated that the TET1 inhibitor Bobcat339 effectively suppresses the expression of DNA repair-related genes by inhibiting their demethylation. These findings suggest that Bobcat339 has the potential to significantly enhance the therapeutic efficacy of sorafenib in eliminating HCC cells in a clinical setting.

## Electronic supplementary material

Below is the link to the electronic supplementary material.

Supplementary Material 1

Supplementary Material 2

Supplementary Material 3

Supplementary Material 4

## Data Availability

The datasets generated during the current study are available in the GEO, http://www.ncbi.nlm.nih.gov/geo/query/acc.cgi?acc=GSE212750. There are still two datasets that have not been uploaded yet which are available from the corresponding author on reasonable request.

## Abbreviations

CCK-8: Cell Counting Kit-8
ChIP: Chromatinimmunoprecipitation
Co-IP: Co-immunoprecipitation
DMSO: Diemthylsulfoxide
EXO1: Exonuclease 1
5-FU: 5-fluorouracil
GEPIA: Gene Expression Profiling Interactive Analysis
GO: Gene Ontology
HA: Hemagglutinin
γH2AX: Phosphorylation of histone H2AX
HCC: Hepatocellular caicinoma
IP: Immunoprecipitation
KEGG: Kyoto Encyclopedia of Genes and Genomes
MST: Mammalian STE20-like protein kinase
RFC5: Replication factor C subunit 5
RT–qPCR: Real-time quantitative polymerase chain reaction
SOR: Sorafenib
TET1: Tet methylcytosine dioxygenase 1
YAP1: Yes-associated protein 1
