# Correction: Epigenetic regulation of DNA repair gene program by Hippo/YAP1-TET1 axis mediates sorafenib resistance in HCC

**DOI:** 10.1007/s00018-024-05448-0

**Published:** 2024-09-30

**Authors:** Chunli Mo, Weixin You, Yipeng Rao, Zhenping Lin, Shuai Wang, Ting He, Huanming Shen, Xun Li, Rui Zhang, Boan Li

**Affiliations:** 1 State Key Laboratory of Cellular Stress Biology, Innovation Center for Cell Signaling Network, School of Life Sciences, Xiamen University, Xiamen, 361100 Fujian China; 2 The First Affiliated Hospital , of Xiamen University, Xiamen, 361100 Fujian China; 3 Department of Laboratory Medicine The First Affiliated Hospital, School of Medicine, Xiamen University, Xiamen, 361003 Fujian China; 4 Xiamen Cell Therapy Research Center, The First Affiliated Hospital, School of Medicine, Xiamen University, Xiamen, 361003 Fujian China; 5 Center for Precision Medicine, The First Affiliated Hospital of Xiamen University, Xiamen, China

**Cellular and Molecular Life Sciences (2024) 81:284**

10.1007/s00018-024-05296-y

In this article, Chunli Mo and Weixin should have been denoted as equally contributing authors.

The original article has been corrected.

