# Supplementary material for: Epigenetic regulation of DNA repair gene program by Hippo/YAP1-TET1 axis mediates sorafenib resistance in HCC
Source: Cell Mol Life Sci. 2024 Jul 5;81(1):284. doi: 10.1007/s00018-024-05296-y (PMC11335208; doi:10.1007/s00018-024-05296-y)
Supplement: Supplementary file 1 — Supplementary Material 1 [file 18_2024_5296_MOESM1_ESM.docx]

**Graphical Abstract:**

**
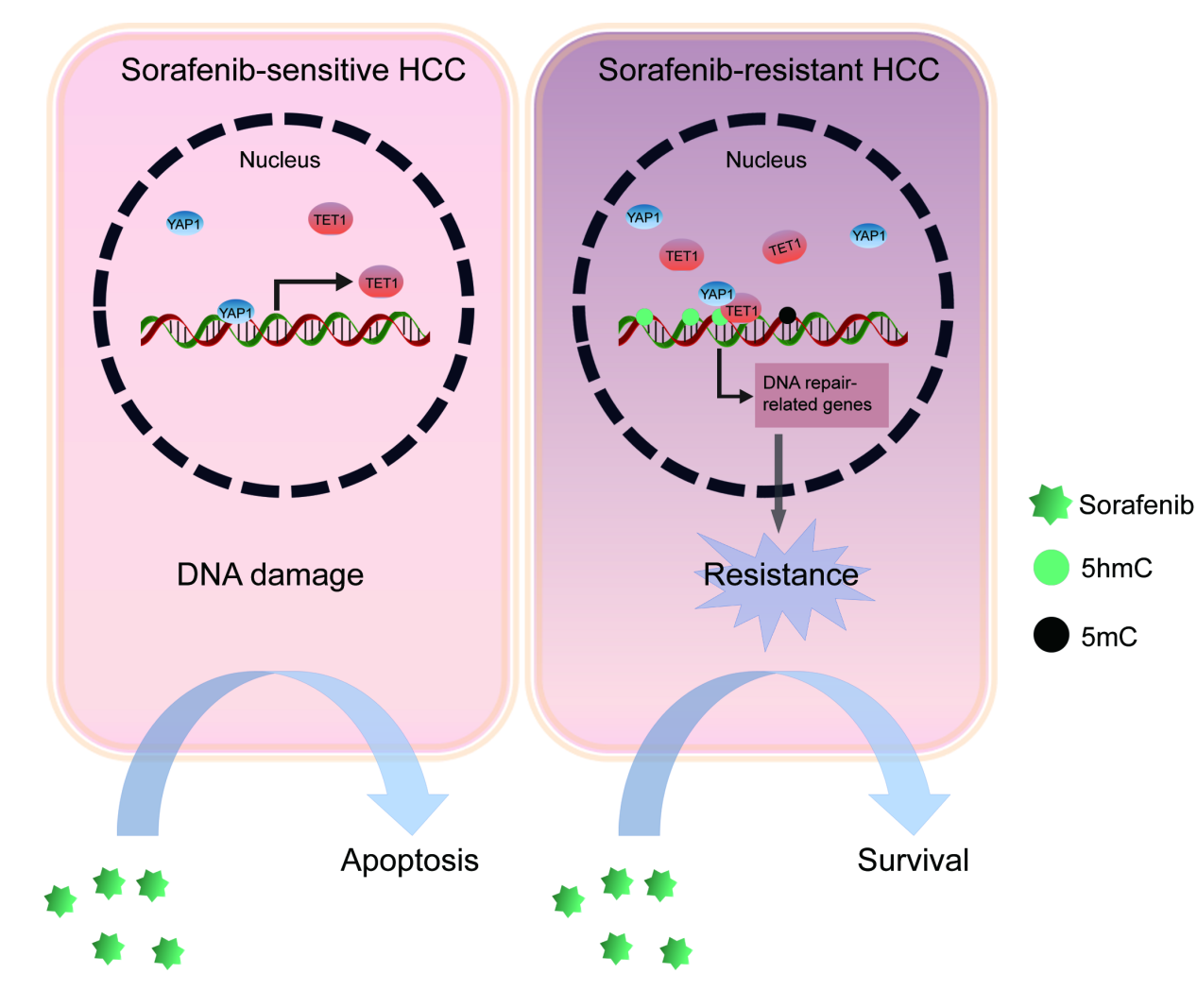
**

In sorafenib sensitive HCC cells, TET1 is transcriptional regulated by YAP1. For long-term stimulation of sorafenib, this transcriptional regulation is significantly enhanced. The enhanced TET1 protein was recruited by YAP1 to the promoter of DNA damage repair-related genes and changed the methylation status of these genes, which ultimately led to the sorafenib resistance.
