# Supplementary material for: Epigenetic regulation of DNA repair gene program by Hippo/YAP1-TET1 axis mediates sorafenib resistance in HCC
Source: Cell Mol Life Sci. 2024 Jul 5;81(1):284. doi: 10.1007/s00018-024-05296-y (PMC11335208; doi:10.1007/s00018-024-05296-y)
Supplement: Supplementary file 2 — Supplementary Material 2 [file 18_2024_5296_MOESM2_ESM.doc]

Supporting information for

**Epigenetic regulation of DNA repair gene program by Hippo/YAP1-TET1 axis mediates sorafenib resistance in HCC**

Chunli Mo,^1,2#^ Weixin You,^1#^ Yipeng Rao,^1^ Zhenping Lin,^1^ Shuai Wang,^1,2^ Ting He,^1^ Huanming Shen,^1^ Xun Li,^2*^Rui Zhang,^2*^ and Boan Li^1*^

**Figure.S1.**


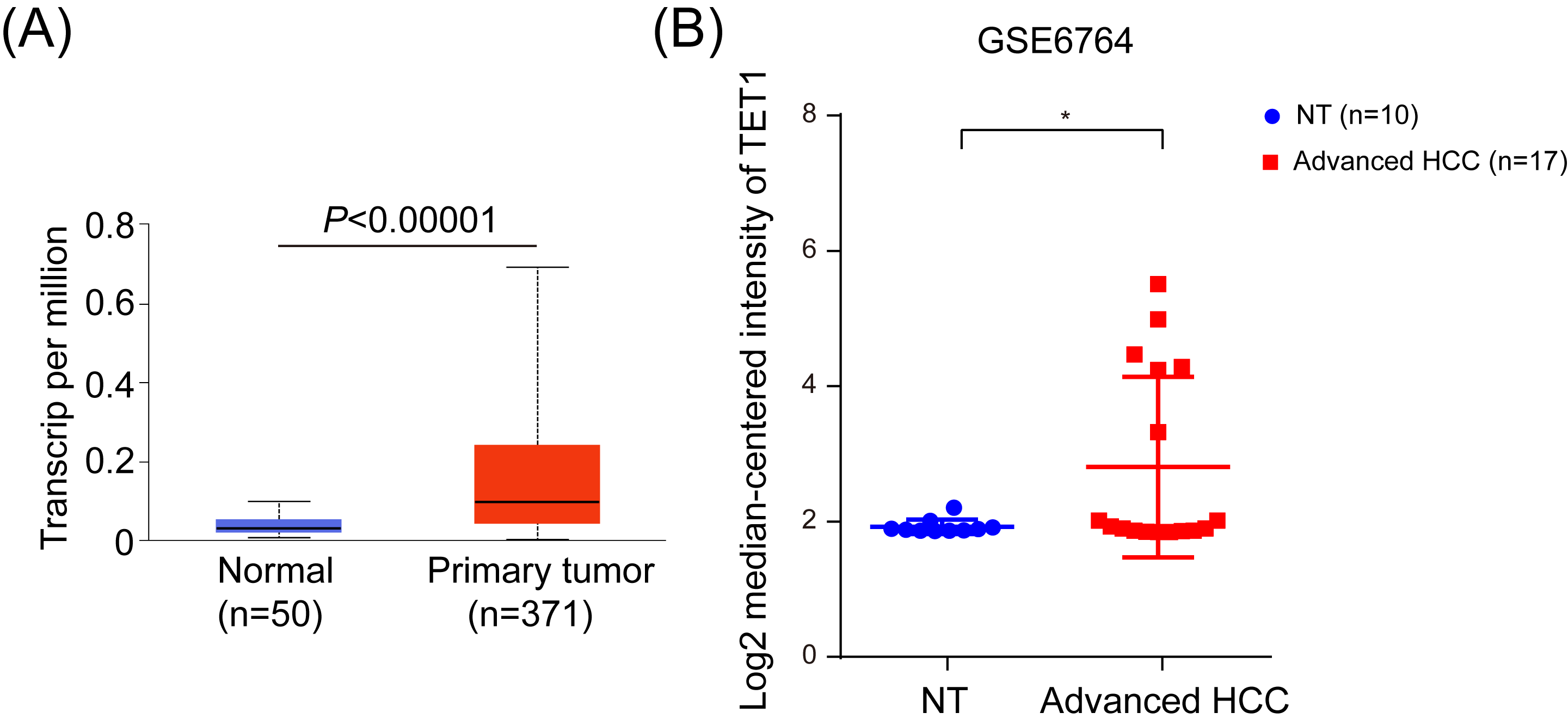


**Figure S1.** **TET1 expression is upregulated in HCC tissues and predicts a poor clinical outcome. (A)** The gene expression of TET1 in HCC samples from GEPIA database (*P* value is shown in the graph). **(B)** The mRNA level of TET1 in 17 advanced HCC and 10 non-tumor livers revealed using the GSE6764 datasets.

**Figure.S2.**


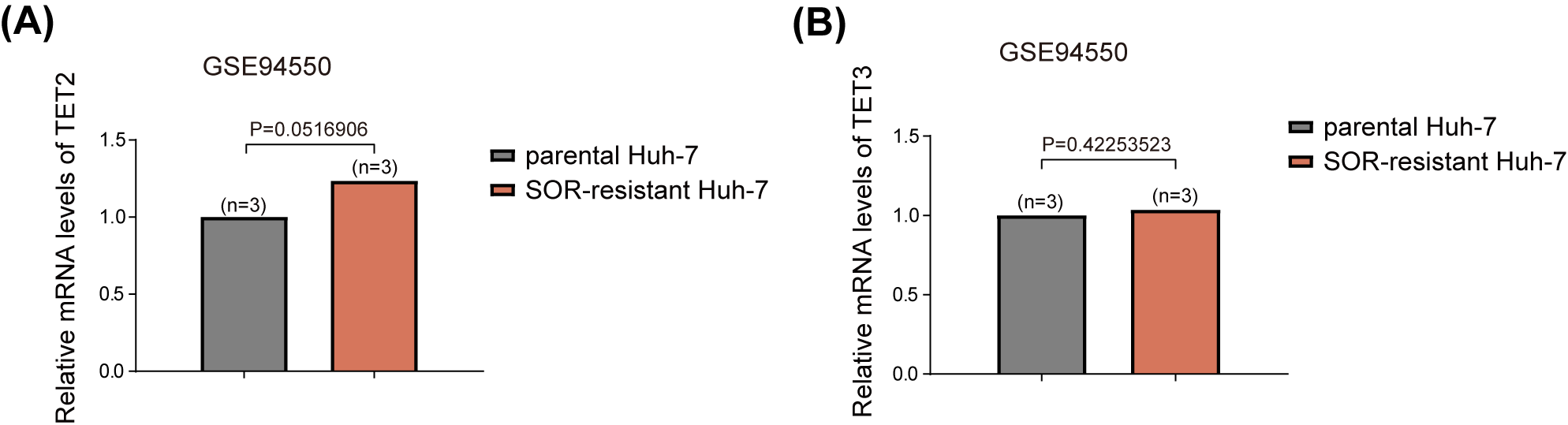


**Figure S2. The mRNA expression levels of TET2 and TET3 were no significant changes in sorafenib-resistant HCC cells. (A)** The mRNA level of TET2 in parental and sorafenib-resistant Huh-7 cells of GSE94550 dataset. **(B)** The mRNA level of TET3 in parental and sorafenib-resistant Huh-7 cells of GSE94550 dataset. Abbreviation: ns, non-significant.

**Figure.S3.**


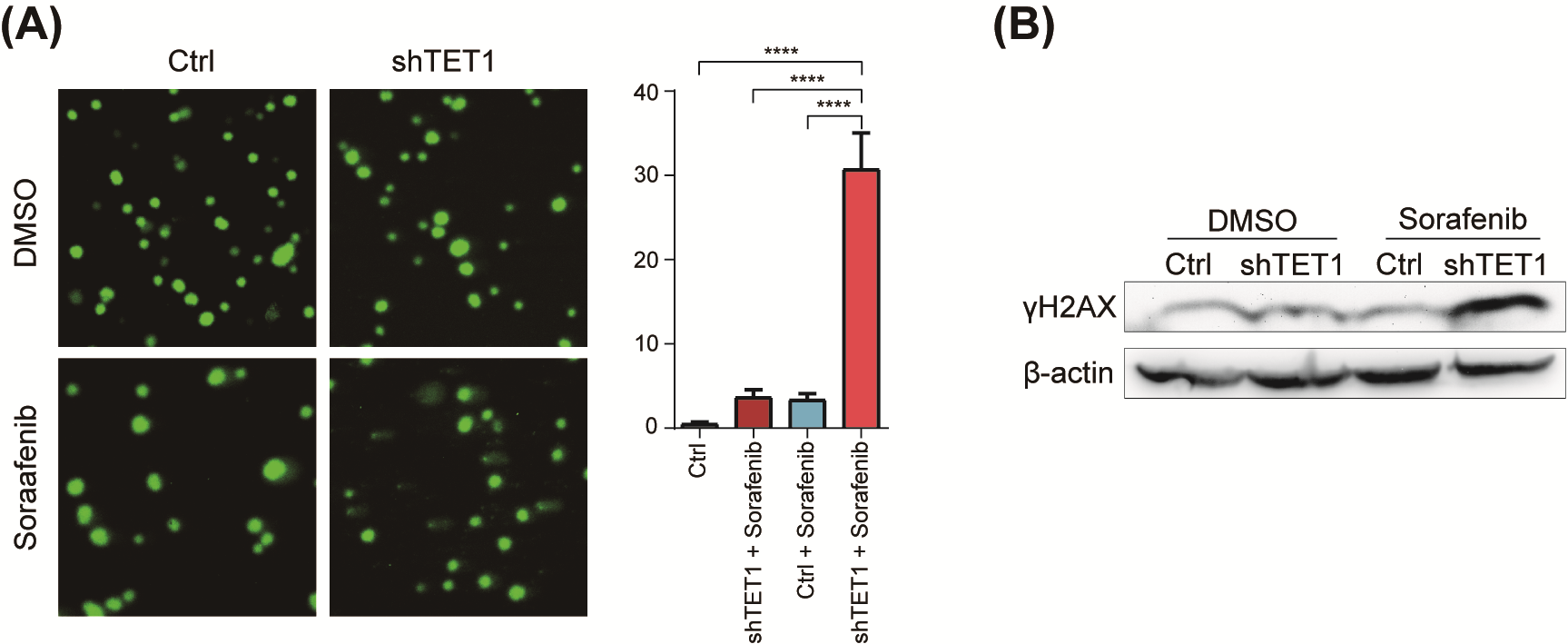


**Figure S3. Inhibition of TET1 increased DNA damage in HepG2 cells treated with sorafenib. (A)** Ctrl and TET1 knockdown HepG2 cells were treated with a nontoxic dose of 2 μM sorafenib. DNA damage were measured by alkaline comet assays, representative comet tails were shown (left), the percentage of DNA in the comet tail were summarized from at least 50 cells (right), statistical analysis was performed using GraphPad software. **(B)** γH2AX protein level of HepG2 cells was measured by western blotting. Data are expressed as the means ± SD of three independent experiments. ****P < 0.0001.

**Figure.S4.**

**Figure S4. Detection of sorafenib resistance in HCC cells. (A)** The sorafenib resistance of Huh-7 cells were determined by Flow cytometry with Annexin V and propidium iodide (PI) double staining. **(B)** The sorafenib resistance of HepG2 cells were determined by Flow cytometry with Annexin V and propidium iodide (PI) double staining. Data are expressed as the means ± SD of three independent experiments. ***P* < 0.01; ****P* < 0.001. **(C)** The protein level of TET family was measured by western blotting.

**Figure.S5.**


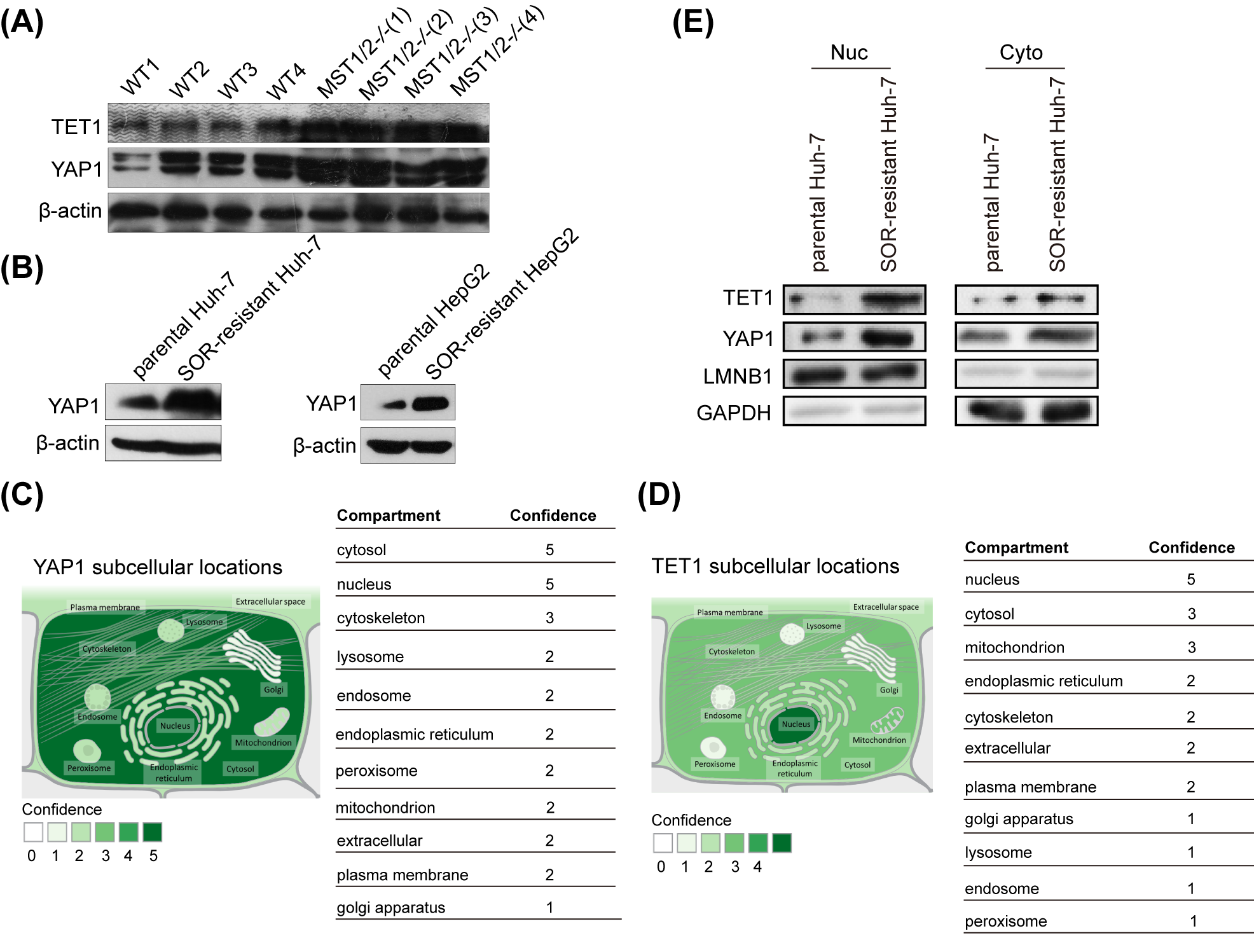


**Figure S5. Treatment with sorafenib affects the subcellular location of YAP1 and TET1 proteins. (A)** The protein levels of TET1 and YAP1 in WT and MST1/2-/- tumor tissues were measured by Western blotting. **(B)** YAP1 protein was upregulated in sorafenib-resistant HCC cells compared to parental cells. **(C)** The YAP1 subcellular location on the website (<https://www.genecards.org/>). **(D)** The TET1 subcellular location on the website (<https://www.genecards.org/>). **(E)** Nuclear plasma separation experiment of parental and sorafenib-resistant Huh-7 cells.

**Figure.S6.**


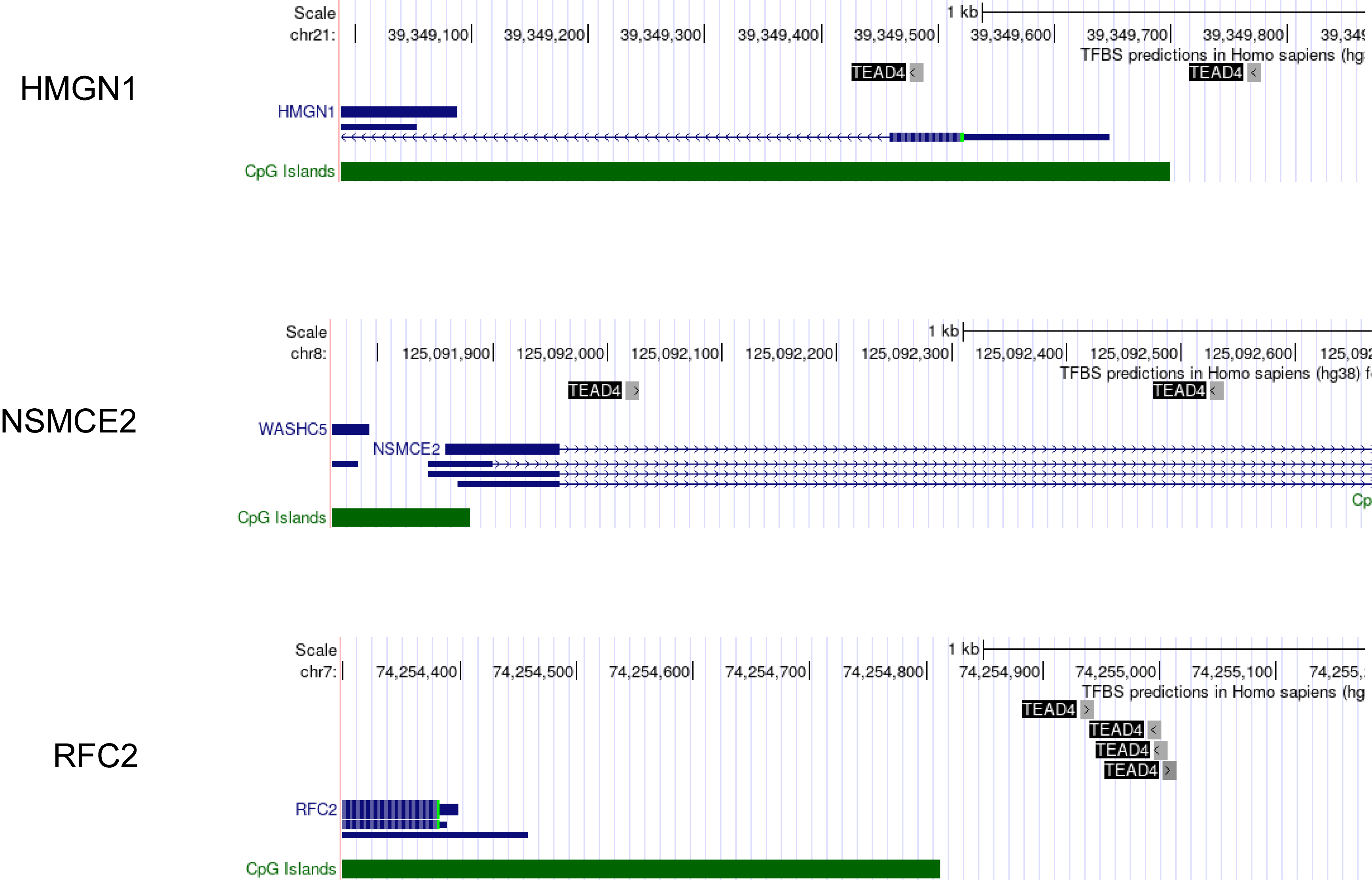


**Figure S6. The predicted binding sites of YAP1/TEAD4 near CpG islands on the enhancer/promoter of DNA repair-related genes.** The visualization of three typical examples as shown by the UCSC genome Browser.

**Figure.S7.**


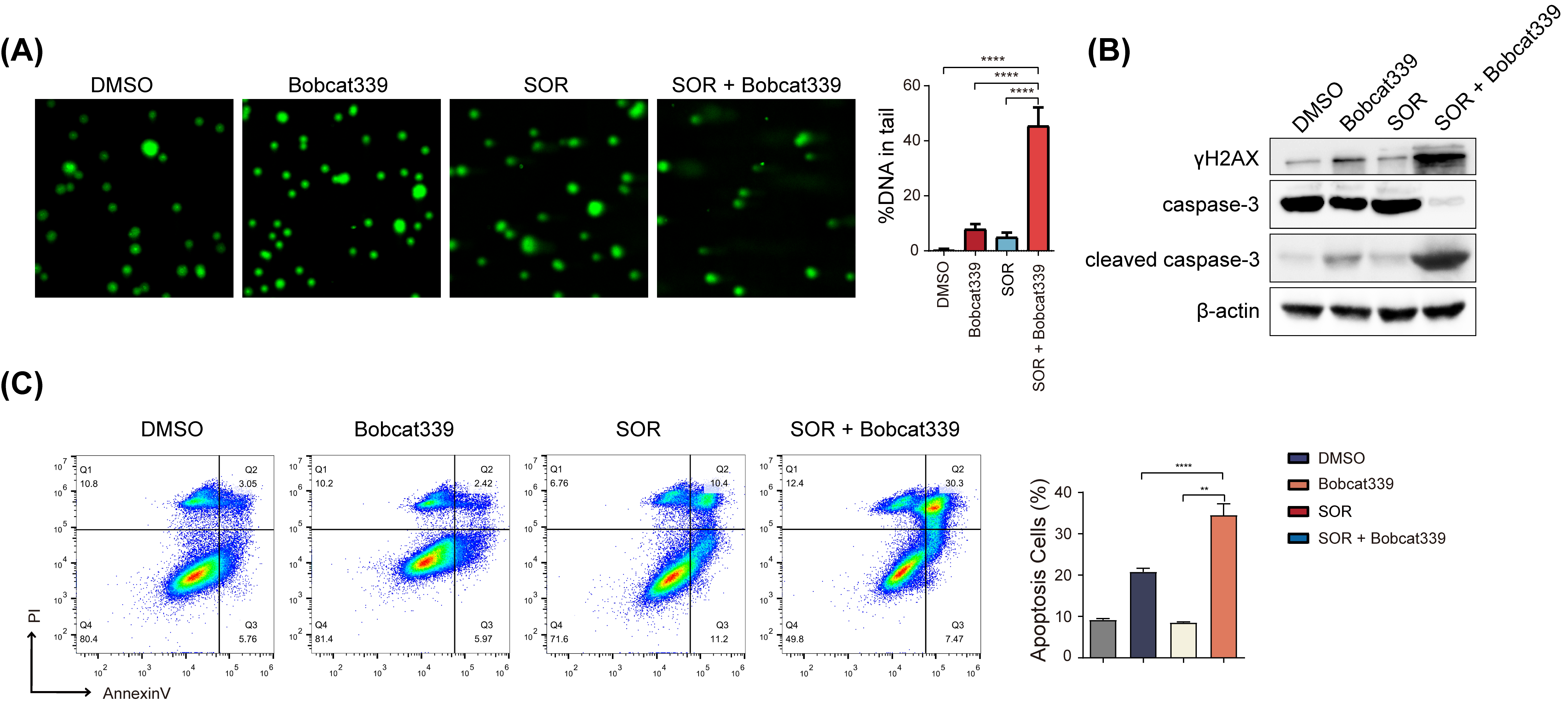


**Figure S7. TET1 inhibitor Bobcat339 could significantly induce DNA damage in combination with sorafenib in HepG2 cells. (A)** The comet assay of HepG2 cells showed that DNA damage was more severe when sorafenib and the TET1 inhibitor Bobcat339 were used in combination, representative comet tails were shown (left), the percentage of DNA in the comet tail were summarized from at least 50 cells (right), statistical analysis was performed using GraphPad software. **(B)** Western blotting showed that the protein levels of the apoptosis markers caspase-3 and cleaved caspase-3 and the DNA damage marker γH2AX in HepG2 cells were sharply increased in the combination therapy group. **(C)** Flow cytometry analysis showed that combination therapy significantly induces apoptosis of HepG2 cells. Data are expressed as the means ± SD of three independent experiments. ***P* < 0.01; *****P* < 0.0001.

**Figure.S8.**


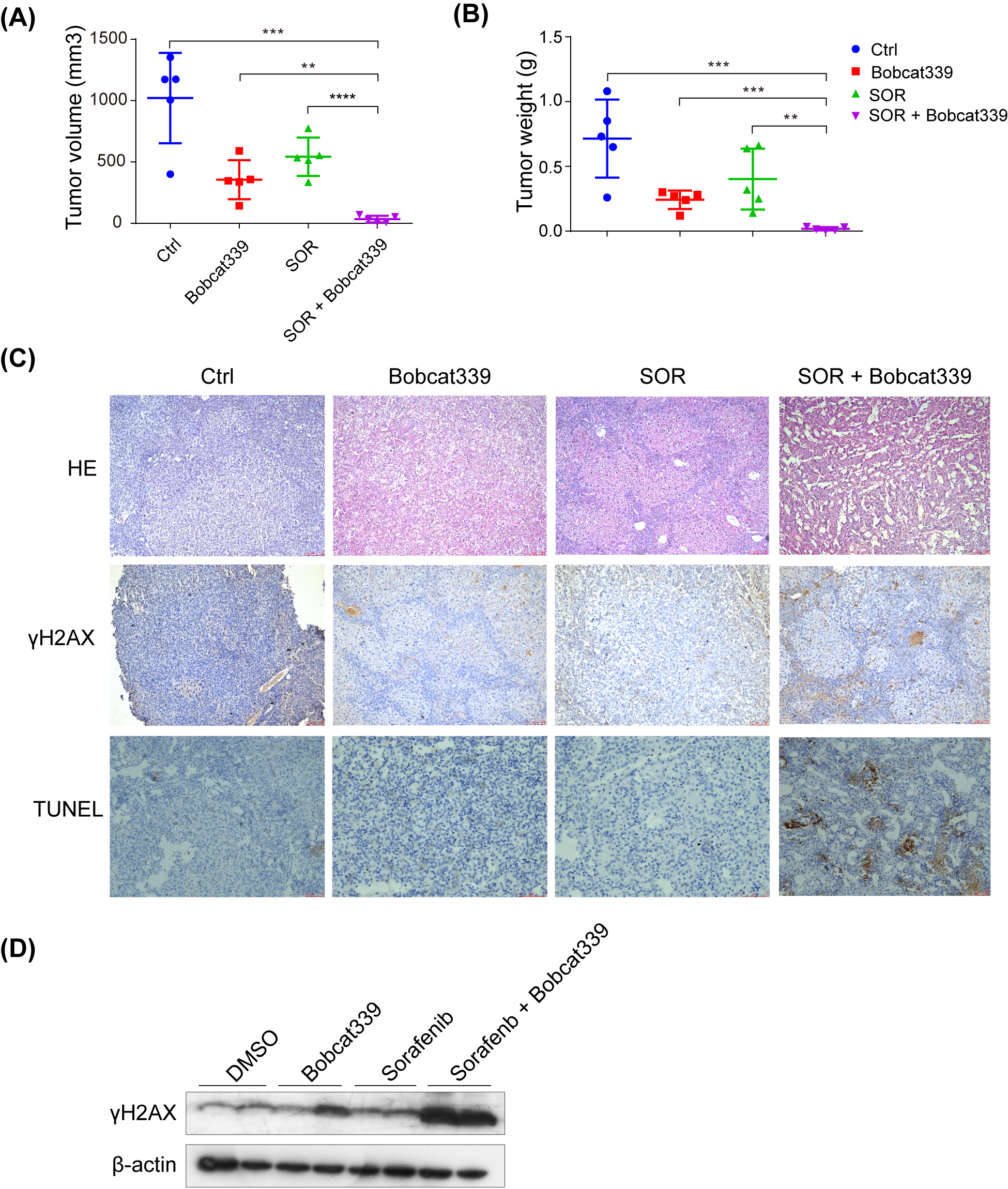


**Figure S8. TET1 inhibitor Bobcat339 combined with sorafenib could significantly induce DNA damage in xenograft model and mst1/2 mutant HCC model.** Nude mice were subcutaneously injected with sorafenib-resistant Huh-7 cells and randomly divided into four groups, treated with sorafenib alone, Bobcat339 alone, sorafenib and Bobcat339, or vehicle controls, comparison tumor volume **(A)** and tumor weight **(B)** among the four mice groups. Error bars represent the means ± SD from n = 5 mice per group. *P < 0.05; **P < 0.01; ***P < 0.001; ****P < 0.0001. **(C)** Immunohistochemistry and TUNEL staining were performed on subcutaneous tumor tissues. Scale bar, 100 μm. **(D)** DNA damage maker γH2AX protein level was increased of the combined treatment group.

**Figure.S9.**

**
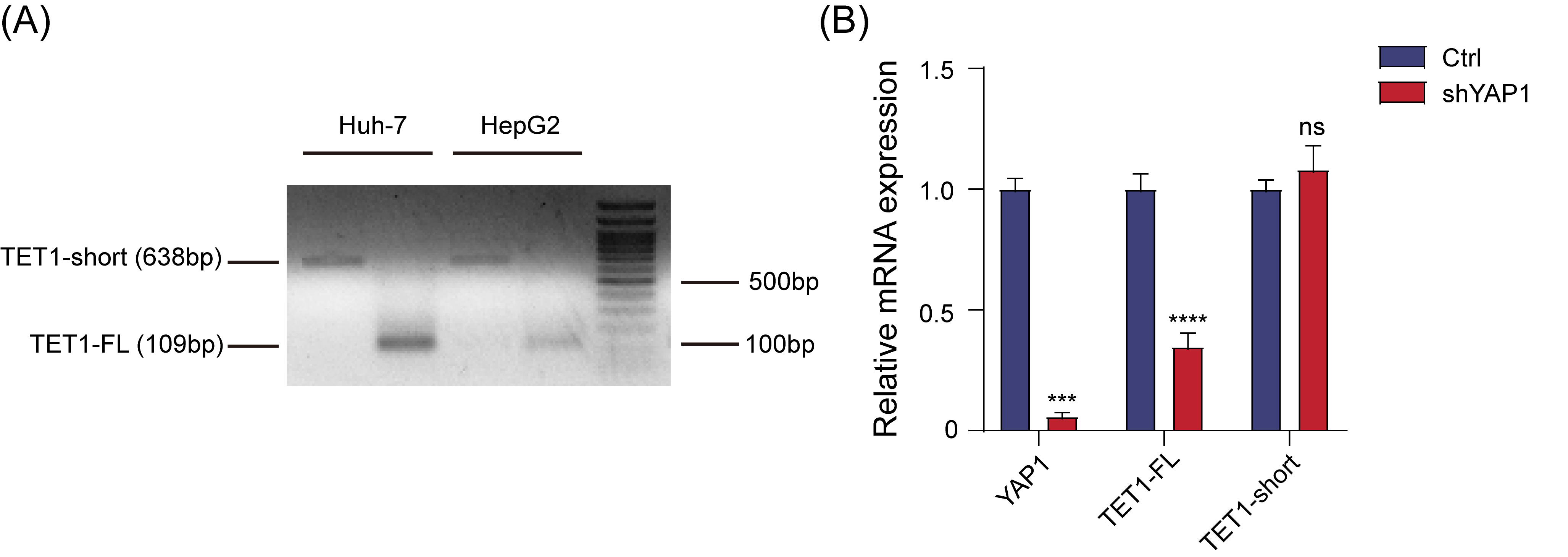
**

**Figure S9. Expression pattern of TET1 isoforms in HCC cell lines. (A)** PCR amplification of TET1-FL and TET1-short in Huh-7 and HepG2 cDNA. PCR products were run on an agarose gel, TET1-short products are observed at 638bp and TET1-FL products are observed at 109bp. (B) qPCR results of different TET1 isoforms in ctrl and shYAP1 Huh-7 cells.

**Figure.S10.**

**
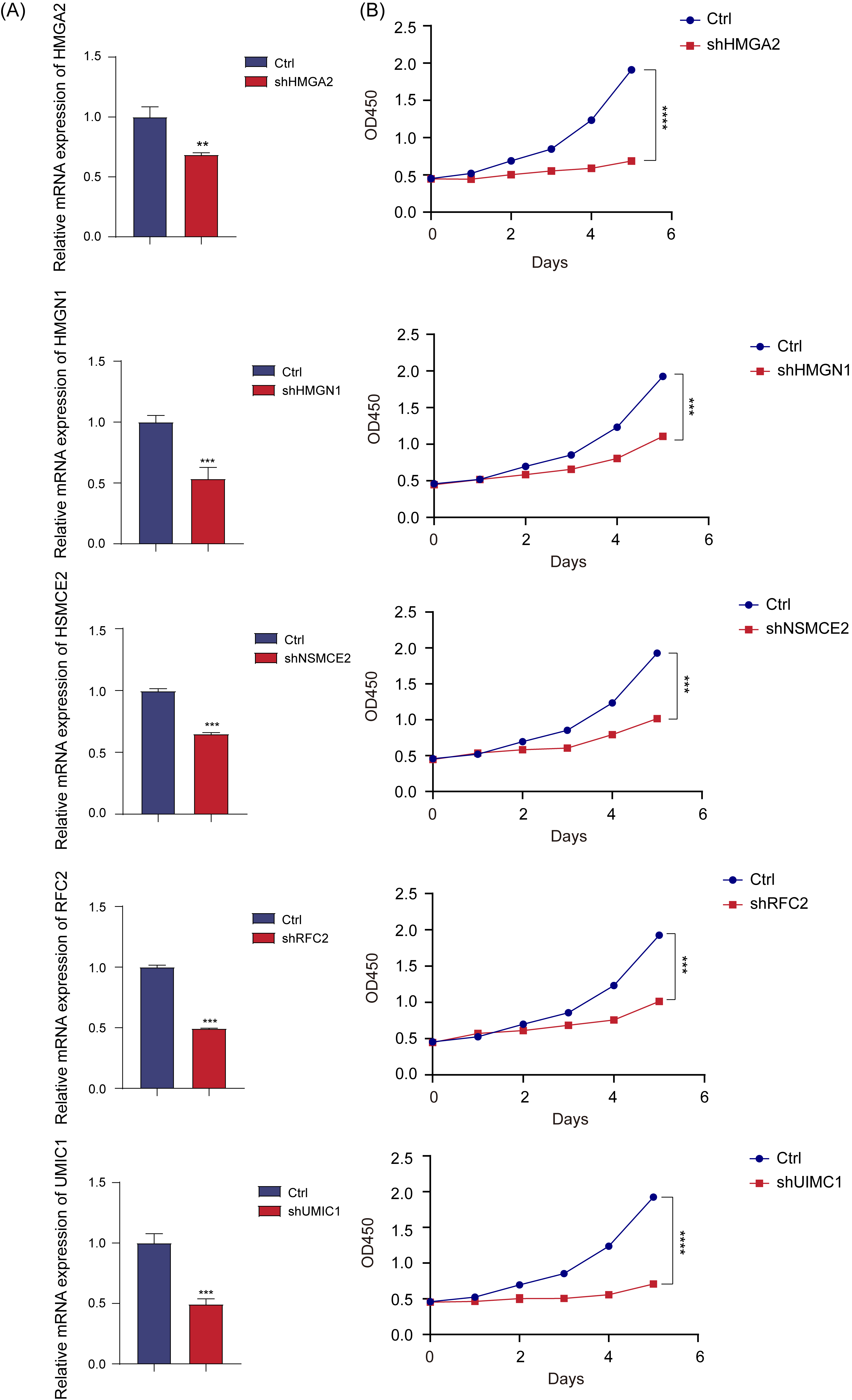
**

**Figure S10. Five key genes of DNA repair affect the proliferation of HCC cells treated with sorafenib.** (A) The mRNA level of the five key genes were measured by RT-qPCR. (B) Cell proliferation of sorafenib-resistant Huh-7 cells with ctrl and knockdown of the five key genes were detected by CCK-8.

Supplementary Table 1:

| **PCR primers used for ChIP-PCR** | | |
| --- | --- | --- |
| **Gene** | **Forward primer (5’ to 3’)** | **Reverse primer (5’ to 3’)** |
| RFC5 | CTTTGAGCCAGGCAATGTTTC | TGTGCATCTGCTGTTCCGTTA |
| XPA | GGCCCAGATCCAAACCCAC | CTTGCTCCAAGGCTGATATGC |
| EXO1 | GGAAAAGAACCCAGCGTGAAC | AAGTTGCTCAGTCCCCCTCG |

Supplementary Table 2:

| **PCR primers used for RT-qPCR** | | |
| --- | --- | --- |
| **Gene** | **Forward primer (5’ to 3’)** | **Reverse primer (5’ to 3’)** |
| POLA1 | AGAAGCTCGCAGTGACAAAAC | AGGTGGTGGAGTTATTTGAGGT |
| CETN2 | GCATCAAGTTCTCAGCGAAAAAG | CCATCCGCATCGAAAAGATCAA |
| POLD3 | ACCAACAAGGAAACGAAAACAGA | GGTTCCGTGACAGACACTGTA |
| SSBP1 | TGAGTCCGAAACAACTACCAGT | CCTGATCGCCACATCTCATTAG |
| PTTG1 | ACCCGTGTGGTTGCTAAGG | ACGTGGTGTTGAAACTTGAGAT |
| MSH6 | TCATCCGCGAGAAAGGGAAAT | ATCTGCACGTTGCATTGCTCT |
| CHEK1 | ACTTACTGCAATGCTCGCTGG | TTGAGGGGTTTGTTGTACCATC |
| MLH1 | CAACAAGTCTGACCTCGTCTTC | CCGGGAATCTGTACGAACCAT |
| CUL1 | GGTTCGCCGTGAATGTGAC | CCCCAATTCCACGTAAGACTGT |
| SSRP1 | TGACTACAAGATCCCCTACACC | GAGTTTGGCCTTGCTTGATTG |
| TP53BP1 | CTCCAGACGCACAAAGAAAATCC | ACCTGACTGATGGAACCACAT |
| E2F5 | TGGCAACTCAAAATCTGCCTG | TTGTAGTCATCTGCCGGGGTA |
| TRIP13 | ACTGTTGCACTTCACATTTTCCA | TCGAGGAGATGGGATTTGACT |
| TET1 | TTGGAGCAAGTGGTAGCCATAGA | TTTGGGTCTTGGAGGTCTTTTCT |
| YAP1 | TATCAATCCCAGCACAGCAAAT | TAGGTGCCACTGTTAAGGAAAGG |
| 18S | GTCTGTGATGCCCTTAGATG | AGCTTATGACCCGCACTTAC |
| TET1-FL | GCGCGAGTTGGAAAGTTTG | GCTCAGTCACACAAGGTTTTGG |
| TET1-short | TTGAAGCCTCCTGTGATTTCG | GGGGCCTCTTGTTTTCCTTTA |

**Supplementary Table 3:**

| **shRNA sequence information** | |
| --- | --- |
| shTET1(1) | CCCAGAAGATTTAGAATTGAT |
| shTET1(2) | GCAGCTAATGAAGGTCCAGAA |
| shYAP1(1) | GCCACCAAGCTAGATAAAGAA |
| shYAP1(2) | CAGGTGATACTATCAACCAAA |

**Supplementary Table 4:**

| **List of Antibodies** | | |
| --- | --- | --- |
| Antibody | Catalog Number | Company |
| TET1 | GTX124207 | GeneTex |
| YAP1 | A1002 | ABclonal |
| γH2AX | 9718 | Cell Signaling Technology |
| Caspase-3 | 19677-1-AP | Proteintech |

**Supplementary Table 5**:

| **List of Reagents** | | |
| --- | --- | --- |
| Reagent | Catalog Number | Company |
| Sorafenib | HY-10201 | MedChemExpress |
| Bobcat339 hydrochloride | HY-111558A | MedChemExpress |
| Olaparib | HY-10162 | MedChemExpress |
| cell counting kit-8 | K1018 | APEXBIO |
| GoldView I | G8140 | Solarbio life sciences |
| One-Step RT-PCR mix kit | G490 | Abmole |
